# Good news reduces trust in government and its efficacy: The case of the Pfizer/BioNTech vaccine announcement

**DOI:** 10.1371/journal.pone.0260216

**Published:** 2021-12-09

**Authors:** Shaun P. Hargreaves Heap, Christel Koop, Konstantinos Matakos, Aslı Unan, Nina Weber

**Affiliations:** Department of Political Economy, King’s College London, London, United Kingdom; University of Edinburgh, UNITED KINGDOM

## Abstract

The announcement of Pfizer/BioNTech’s COVID-19 vaccine success on November 9, 2020 led to a global stock market surge. But how did the general public respond to such good news? We leverage the unexpected vaccine announcement to assess the effect of good news on citizens’ government evaluations, anxiety, beliefs and elicited behaviors in the US and the UK. While most outcomes were unaffected by the news, trust in government and elected politicians (and their competency) saw a significant decline in both countries. As the news did not concern the governments, and the governments did not have time to act on the news, our results suggest that the decline of trust is more likely explained by the psychological impact of good news on reasoning style. In particular, we suggest two possible styles of reasoning that might explain our results: a form of motivated reasoning and a reasoning heuristic of relative comparison.

## Introduction

Pfizer/BioNTech’s announcement on November 9, 2020 that its COVID-19 vaccine was, according to its own clinical trials, 90% effective triggered a global surge in stock markets. Actors in the markets plausibly adjusted their beliefs about future corporate earnings as a result of the announcement; noticeably so for those companies that had been most affected by the COVID-19 lockdowns as their share prices rose the most [1]. In this paper, we are concerned with whether the general public also adjusted their beliefs, attitudes or behavior in response to this good news. We answer this question by leveraging the vaccine news as an unexpected event in nation-wide surveys in the US and the UK. Our main result is that, although, as Pfizer suggested, ‘this was a great day for science and humanity’, it was *not* a great day for government. Trust in government and elected politicians (and their competency) fell in both countries in response to the vaccine announcement. Or in other words, positive news caused a sizeable reduction in political trust.

This is a new as well as a surprising result. It is new because, to our knowledge, the influence of positive news on trust in government and elected politicians has not been studied before. There is evidence on the effect of negative news. It is well known, for example, that at the outset of the COVID-19 pandemic, trust in government and support for incumbents rose [2–6]. This rise has been variously interpreted as a ‘rally round the flag’ and a response to COVID-19-related anxiety. There are similar results for the H1N1 flu [7] and for other disasters like tornados and floods, though the results are not always as clear [8]. Good news, on the other hand, has not been studied in the same way. Several studies have shown that the release of (good or bad) municipal audit outcomes or information about political corruption affects citizens’ attitudes, voting behavior and tax payments [9–11]. Yet, in these studies, the (good or bad) news is directly related to government and politicians’ performance. By contrast, we are interested in identifying the general effect of ‘good news’, especially when seemingly unrelated to government actions and performance.

The result is surprising because, in contrast with the literature on the determinants of government trust where it is government actions and efficacy that cause changes in trust [12, 13], we find that trust in governments depends on something other than their actions, at least during COVID-19-like episodes. There is a large literature in psychology on how positive (negative) news can cause positive (negative) affect or mood changes in individuals with the result that individuals assess events and behave differently and usually in a manner congruent with their mood change [14]. However, with the vaccine announcement, there was no congruence: the evaluation of government moved in the opposite direction (i.e., negative) to the likely change in affect from the (positive) announcement. Indeed, the same discordance was observed after the onset of COVID-19 bad news when trust in government improved. Yet, whereas that initial improvement in the evaluation of government could have been related to the actions of government in the face of the pandemic (e.g., imposition of strict lockdowns and the introduction of other restrictive measures to stop the spread of the virus), there was no change in our experiment in the evidential base on government actions from which to form judgments about trust and competence. The government and elected politicians had no time to do anything material, except applaud the discovery of an effective vaccine.

We draw instead on the strand of the psychology literature on emotions where mood has been found to affect reasoning style, with good news tending to encourage top-down heuristic reasoning with little attention to detail [15]. Thus, we hypothesize two possible reasoning style mechanisms. First, there is a form of motivated reasoning. The COVID-19 pandemic constituted a major new source of uncertainty and anxiety for many people [16]. To maintain a sense of self-efficacy during those challenging times, they needed to pin their hopes on something that could contain the new source of uncertainty and anxiety [17]. As a result, they adjusted their beliefs accordingly. Governments are a possible agent for controlling COVID-19 uncertainty and trust in government is a sufficiently subjective belief to admit adjustment. This might explain why trust in governments first rose (in what is known as a ‘rally round the flag’ effect). But when the vaccine was announced, people had something else to pin their hopes on. Consequently, their need for trust in government declined. One can think of it as a ‘reset’ in people’s beliefs about governments, returning them to their pre-pandemic levels, once the announcement signaled that we would be soon reaching the end of the current crisis.

Second, the reasoning heuristic may have been that of relative comparison. The vaccine announcement did not concern government efficacy; hence, its competence in absolute terms should not have changed. Yet, the same is not true in relative terms. In particular, the vaccine news shed a positive light on the competence of scientists. By comparison, governments’ competence appeared rather bleak. This, in turn, may account for the decline in trust in government and politicians. Though we cannot directly test these two mechanisms, our evidence suggests that both may have been at work in the aftermath of the good vaccine news.

Our results have important implications for government efficacy and performance as the main explanatory factors behind the scarring effects of pandemics and other crises in general [13]. Since we find that such effects are more likely to arise from the operation of psychological reasoning mechanisms as opposed to an assessment of governments’ own actions and decisions, our results are put in sharp contrast with existing theories on the determinants of political trust.

### Connection to the literature and contribution

Our results contribute to the literature in two main ways. First, there is a large literature in psychology and economics on how positive (negative) news can cause positive (negative) affect or mood changes in individuals, with the result that they behave and process information differently in a range of other social settings. These studies focus, for instance, on mood and the dictator game [18–20], mood and risk preferences [21–24], and mood and public goods and trust games [15, 25–27]. Positive affect has been studied less often in this literature than negative affect. Though our study was conducted in the particular context of the COVID-19 pandemic, and cannot readily be extrapolated, the results suggest caution in extending findings on negative affect to the domain of positive affect. We find little evidence that the vaccine announcement affected key elements of social capital (i.e., generalized trust, risk preferences, discount rates, gifts in a dictator game or the propensity to contribute to charity).

Second, unlike the extant literature on the determinants of trust in government, which mostly relies on informational theories of government efficacy as well as performance and accountability, we find that trust was unrelated to the actual actions of government. We propose that this might be understood by the way that mood affects the style of reasoning. On the one hand, the good news may have encouraged a form of motivated reasoning [28], whereby citizens adjusted their prior beliefs, placing their hope in medicine and scientific discovery instead of government. We present some additional evidence in support of this interpretation. Insofar as this psychological mechanism was at work, our results have the further implication that in the apparent association between political trust and compliance with policies, it is not trust that determines compliance; rather, the exogenous shocks to uncertainty and the anxiety in decision-making associated with it cause both to move.

On the other hand, citizens may have relied on the heuristic of relative comparison. Although the behavior of government did not change in absolute terms, it did relative to that of scientists, with government looking, in comparison, rather ineffectual. We also present some evidence in support of this explanation, with evaluations of both generic and specific government competency being negatively affected by the news.

The paper proceeds as follows. In the next section we present our research design, sampling and econometric methods. Section 3 presents our main results, while Section 4 concludes with some discussion over the possible mechanisms and implications of our findings.

## Materials and methods

**Fig 1:**
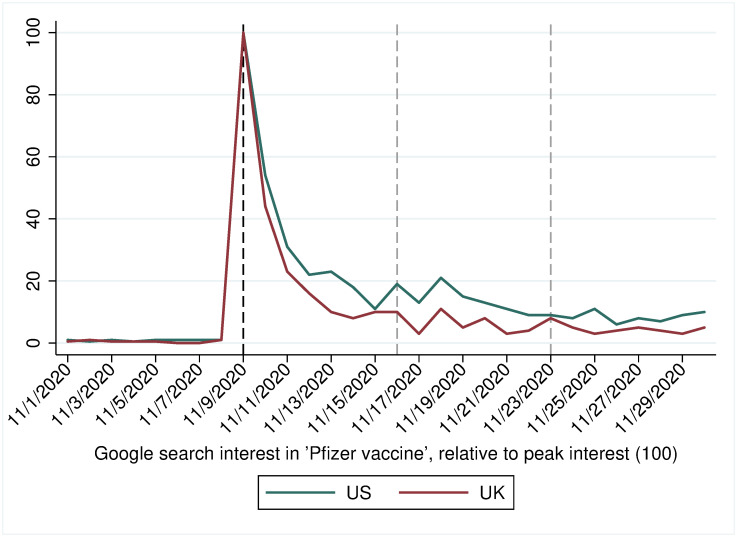
Public interest in the vaccine. Search interest in ‘Pfizer vaccine’ in the US and UK, as measured by Google Trends. The reference lines are for the announcement of the vaccine developed by Pfizer/BioNTech (in black), and the later announcements by Moderna and Oxford University/AstraZeneca, respectively (both in gray).

To determine the impact of the vaccine announcement, we added a wave to an online survey that we had conducted in the UK and the US the day and early morning before the vaccine was announced (see S1 and S2 Tables for details on the quota-based sample). The research was registered by KCL’s Ethics Committee as MRSP-19/20–18237. While the original survey was pre-registered via https://osf.io/qtes9/files/, we had not listed any vaccine-related hypotheses as the vaccine news was unanticipated. We had recruited our initial target sample by the morning of November 9, 2020 (before the vaccine announcement), and therefore added a survey wave by recruiting a new sample from November 19 to 22 based on the same quotas. We could leverage the vaccine announcement to identify the effect of positive news on the general public—with respondents in the first and second waves serving as, respectively, control and treatment groups [29]—because the news was unexpected and attracted an enormous amount of public interest (see Fig 1). More specifically, while some vaccine news was expected in late 2020, the timing was unknown, and the high level of effectiveness of the vaccine was wholly unforeseen. Furthermore, the event was extremely salient and covered in the news for weeks (see S1 Fig). Hence, we can assume that respondents were exposed to the vaccine announcement. Nonetheless, as a robustness check, we rerun our analyses on the subset of respondents that consume a lot of news (see S9 Table). By relying on nation-wide surveys and a naturally occurring event, the external validity of the results is higher than in controlled (field and especially laboratory) experiments.

### Sample and quasi-experimental design

We conducted our online experiment using Qualtrics for the design and Prolific Academic for the recruitment of participants. Prolific Academic is a web-based panel with about 100,000 participants, mainly in the US and UK. We preferred it over mTurk as its participants have been found to pay more attention and provide responses of higher quality [30]. Our quota-based sample was recruited between November 8 and November 22, 2020. To generate samples for our two countries, we used the US Current Population Survey [31], the 2011 UK Census [32], and Scotland’s 2011 Census [33]. Northern Ireland was excluded from the survey. We created a total of 170 subgroups weighted based on age, gender, region and work status. S1 and S2 Tables report the total number of respondents per subgroup in the US and UK, respectively. The average completion time of the full survey was 19.48 minutes and respondents earned on average £2.50 for their participation. The full survey instrument is included in S3 Appendix.

The vaccine announcement did not coincide with the occurrence of any other major event in the two countries during the period of our survey: the results of the US presidential election of November 3 were known. While uncertainty remained in some states throughout the survey period, all major news outlets (including Fox News) had called the election in favor of Joe Biden, and protests at polling stations occurred in the week preceding our pre-treatment data collection. Indeed, as S2 Fig shows, public interest in the election results (and in electoral fraud) had declined considerably by the time of the vaccine announcement. We would therefore expect our pre-treatment measures of political trust to be at least as negatively affected by the events surrounding the elections as our post-treatment measures, if not more. In other words, the negative effect on trust we document is arguably a lower bound estimate, since the baseline pre-treatment levels of trust were probably already very low. In the UK, a second lockdown had been announced on November 1 and already been implemented from November 5 (see ‘Coincidental events’ in S1 Appendix). Given previous research on the effects of lockdowns [2, 3, 5], one would therefore expect trust in government to have increased rather than decreased in the studied time period. In S1 Appendix, we discuss collateral and coincidental events and how these might affect the validity of our design.

We newly recruited our treatment group based on the same set of quotas as the control group. This led us to consider the assignment of respondents to the two groups ‘as good as random’ [29]. Yet, as studies of unexpected events are not based on full randomization, we performed difference-of-means tests on the demographic composition of the two groups. As our samples are slightly different in some respects—in the UK more than in the US (see S3 Table) –, we present our main models using sample weights (Table 1) as well as entropy balance weights (Table 2). The latter allow the distribution of the covariates among the treatment group to mimic the distribution among the control (target) group so to have a balanced sample [34].

**Table 1:** Treatment effects of the vaccine announcement.

	United States			United Kingdom		
	All respondents	Highly exposed	Risk group	All respondents	Highly exposed	Risk group
**Government assessment**						
Trust in government	-0.143***	-0.206***	-0.040	-0.118***	-0.198**	-0.206**
	(0.051)	(0.065)	(0.101)	(0.016)	(0.071)	(0.084)
	[0.008]	[0.004]	[0.924]	[0.001]	[0.061]	[0.058]
Trust in politicians	-0.176***	-0.297***	-0.225	-0.131**	-0.291**	-0.192
	(0.055)	(0.067)	(0.158)	(0.046)	(0.125)	(0.141)
	[0.007]	[0.001]	[0.924]	[0.019]	[0.061]	[0.072]
Government competency	-0.156**	-0.124	-0.150	-0.157**	-0.213*	-0.272**
	(0.070)	(0.149)	(0.151)	(0.064)	(0.113)	(0.113)
	[0.011]	[0.158]	[0.924]	[0.023]	[0.064]	[0.058]
**Measures of anxiety**						
Concern	0.124**	-0.053	0.081	0.034	0.037	-0.109
	(0.058)	(0.080)	(0.122)	(0.052)	(0.093)	(0.170)
	[0.080]	[1.000]	[1.000]	[0.352]	[0.678]	[0.365]
Economic concern	0.077	0.049	-0.054	-0.088*	-0.110	-0.224**
	(0.050)	(0.090)	(0.087)	(0.041)	(0.081)	(0.083)
	[0.080]	[1.000]	[1.000]	[0.134]	[0.678]	[0.049]
**Beliefs about the world**						
Seriousness	0.044	-0.014	0.057	-0.006	-0.042	-0.023
	(0.035)	(0.071)	(0.077)	(0.044)	(0.051)	(0.083)
	[0.457]	[1.000]	[1.000]	[1.000]	[0.799]	[1.000]
Others follow guidelines	-0.103	-0.184**	-0.071	-0.001	-0.142	0.093
	(0.073)	(0.087)	(0.154)	(0.094)	(0.178)	(0.213)
	[0.457]	[0.133]	[1.000]	[1.000]	[0.799]	[1.000]
Luck vs. effort	-0.029	0.021	0.251	-0.191	-0.426	-1.159*
	(0.172)	(0.323)	(0.370)	(0.244)	(0.357)	(0.611)
	[0.457]	[1.000]	[1.000]	[1.000]	[0.799]	[0.354]
**Elicited behaviors**						
Willingness to pay	0.643	-20.852***	4.605	-11.497**	-8.054	-26.431
	(5.203)	(6.434)	(11.651)	(4.835)	(10.35)	(11.202)
	[1.000]	[0.005]	[0.533]	[0.085]	[0.832]	[0.087]
Willingness to comply	-0.028	-0.126	0.145*	0.001	0.091	0.065
	(0.056)	(0.113)	(0.072)	(0.062)	(0.075)	(0.170)
	[1.000]	[0.157]	[0.117]	[0.975]	[0.832]	[0.551]
**Social capital**						
Patience	-0.078	0.046	0.311	-0.295	-0.182	-0.716
	(0.158)	(0.307)	(0.268)	(0.201)	(0.223)	(0.370)
	[1.000]	[1.000]	[1.000]	[1.000]	[1.000]	[0.695]
Generalized trust	0.041	0.048	0.027	-0.014	-0.073	0.077
	(0.032)	(0.048)	(0.076)	(0.034)	(0.064)	(0.091)
	[1.000]	[0.449]	[1.000]	[1.000]	[1.000]	[0.713]
Risk taking	-0.226	-0.178	-0.464*	-0.043	0.250	-0.019
	(0.137)	(0.215)	(0.269)	(0.173)	(0.203)	(0.304)
	[1.000]	[0.449]	[0.835]	[1.000]	[1.000]	[1.000]
Dictator game sharing	0.027	0.296	-0.004	-0.043	-0.014	-0.401
	(0.140)	(0.174)	(0.262)	(0.131)	(0.234)	(0.286)
	[1.000]	[0.321]	[1.000]	[1.000]	[1.000]	[0.695]
Altruism	0.926	-29.195	22.810	-3.662	13.580	-13.338
	(12.893)	(15.163)	(36.360)	(11.084)	(19.160)	(15.378)
	[1.000]	[0.321]	[1.000]	[1.000]	[1.000]	[0.713]
Observations	1,381	605	448	1,236	457	234

Notes: Each estimate comes from an individual linear or logistic regression. Trust in government ranges from 1–4, trust in politicians and government competency from 1–5 with higher values indicating a more positive assessment. Measures of anxiety range from 1 to 4 with higher values indicating more concern. Seriousness (1–4) captures the perceived seriousness of COVID-19 compared to the flu. Others follow guidelines (1–5) captures the perceived likelihood that others comply with government guidelines. Luck vs. effort (0–10) indicates whether income differences are perceived to result from luck (0) or from effort (10). Willingness to pay ranges from $/£0 to $260/£200 capturing the amount respondent i is willing to pay for a treatment to reduce own mortality from COVID-19. Willingness to comply (1–4) captures the self-reported likelihood to comply with guidelines. For all social capital variables, higher values indicate more patience (0–10), trust (0–1), willingness to take risks (0–10), dictator game sharing (0–10) and altruism (0–1000). Controls include gender, age, political affiliation, education and income. State- and region-clustered standard errors are in parenthesis. Adjusted p-values for multiple hypothesis testing are presented in brackets. Asterisks correspond to significance-levels without adjusted p-values.

*** p<0.01,

** p<0.05,

* p<0.1.

**Table 2:** Treatment effects, with entropy balanced data.

	United States			United Kingdom		
	All respondents	Highly exposed	Risk group	All respondents	Highly exposed	Risk group
**Government Assessment**						
Trust in government	-0.128**	-0.195***	-0.016	-0.104***	-0.162*	-0.180**
	(0.050)	(0.062)	(0.092)	(0.027)	(0.081)	(0.078)
	[0.014]	[0.004]	[1.000]	[0.007]	[0.104]	[0.051]
Trust in politicians	-0.163***	-0.291***	-0.202	-0.119**	-0.229*	-0.169
	(0.051)	(0.068)	(0.146)	(0.053)	(0.124)	(0.149)
	[0.010]	[0.001]	[1.000]	[0.017]	[0.104]	[0.104]
Government competency	-0.142**	-0.127	-0.094	-0.220***	-0.216*	-0.375**
	(0.067)	(0.152)	(0.146)	(0.059)	(0.108)	(0.130)
	[0.020]	[0.157]	[1.000]	[0.007]	[0.104]	[0.051]
**Measures of anxiety**						
Concern	0.130**	-0.032	0.098	0.034	0.045	-0.110
	(0.060)	(0.080)	(0.125)	(0.043)	(0.088)	(0.144)
	[0.073]	[1.000]	[1.000]	[0.288]	[1.000]	[0.299]
Economic concern	0.074	0.051	-0.046	-0.080*	-0.103	-0.153
	(0.047)	(0.088)	(0.085)	(0.044)	(0.085)	(0.089)
	[0.073]	[1.000]	[1.000]	[0.238]	[1.000]	[0.299]
**Beliefs about the world**						
Seriousness	0.040	-0.007	0.046	0.016	-0.012	0.013
	(0.033)	(0.070)	(0.076)	(0.042)	(0.048)	(0.086)
	[0.520]	[1.000]	[1.000]	[1.000]	[1.000]	[1.000]
Others follow guidelines	-0.097	-0.194**	-0.036	-0.005	-0.140	0.036
	(0.070)	(0.086)	(0.153)	(0.099)	(0.177)	(0.238)
	[0.520]	[0.096]	[1.000]	[1.000]	[1.000]	[1.000]
Luck vs. effort	-0.083	-0.010	0.213	-0.187	-0.374	-0.704
	(0.170)	(0.328)	(0.381)	(0.194)	(0.320)	(0.571)
	[0.520]	[1.000]	[1.000]	[1.000]	[1.000]	[1.000]
**Elicited behaviors**						
Willingness to pay	0.323	-19.817***	2.961	-10.198*	-6.249	-24.466**
	(4.912)	(6.312)	(11.495)	(5.611)	(9.726)	(10.377)
	[1.000]	[0.007]	[0.664]	[0.247]	[1.000]	[0.087]
Willingness to comply	-0.034	-0.137	0.139	-0.009	0.091	-0.029
	(0.057)	(0.115)	(0.075)	(0.055)	(0.078)	(0.149)
	[1.000]	[0.137]	[0.169]	[0.777]	[1.000]	[0.738]
**Social capital**						
Patience	-0.067	0.038	0.451*	-0.254	-0.205	-0.698*
	(0.155)	(0.307)	(0.262)	(0.200)	(0.231)	(0.306)
	[1.000]	[0.564]	[0.389]	[1.000]	[1.000]	[0.299]
Generalized trust	0.051	0.063	0.036	-0.003	-0.052	0.051
	(0.032)	(0.149)	(0.079)	(0.033)	(0.068)	(0.082)
	[0.429]	[0.450]	[0.830]	[1.000]	[1.000]	[0.696]
Risk taking	-0.248*	-0.194	-0.445	-0.133	0.197	0.059
	(0.135)	(0.213)	(0.275)	(0.167)	(0.254)	(0.319)
	[0.429]	[0.450]	[0.389]	[1.000]	[1.000]	[1.000]
Dictator game sharing	0.016	0.268	0.015	-0.063	0.073	-0.354
	(0.138)	(0.172)	(0.262)	(0.116)	(0.189)	(0.215)
	[1.000]	[0.450]	[0.830]	[1.000]	[1.000]	[0.356]
Altruism	2.210	-26.837	33.405	-9.161	4.257	-12.172
	(11.531)	(14.782)	(30.034)	(7.908)	(19.640)	(14.705)
	[1.000]	[0.450]	[0.389]	[1.000]	[1.000]	[0.696]
Observations	1,381	605	448	1,236	457	234

Notes: Each estimate comes from an individual linear or logistic regression. Trust in government ranges from 1–4, trust in politicians and government competency from 1–5 with higher values indicating a more positive assessment. Measures of anxiety range from 1 to 4 with higher values indicating more concern. Seriousness (1–4) captures the perceived seriousness of COVID-19 compared to the flu. Others follow guidelines (1–5) captures the perceived likelihood that others comply with government guidelines. Luck vs. effort (0–10) indicates whether income differences are perceived to result from luck (0) or from effort (10). Willingness to pay ranges from $/£0 to $260/£200 capturing the amount respondent i is willing to pay for a treatment to reduce own mortality from COVID-19. Willingness to comply (1–4) captures the self-reported likelihood to comply with guidelines. For all social capital variables, higher values indicate more patience (0–10), trust (0–1), willingness to take risks (0–10), dictator game sharing (0–10) and altruism (0–1000). Controls include gender, age, political affiliation, education and income. State- and region-clustered standard errors are in parenthesis. Adjusted p-values for multiple hypothesis testing are presented in brackets. Asterisks correspond to significance-levels without adjusted p-values.

*** p<0.01,

** p<0.05,

* p<0.1.

### Data and measurement

We measure generalized trust, trust in government, trust in politicians and government competence using questions that are identical to those asked in surveys conducted by the American National Election Studies [35]. We measure COVID-19-induced anxiety by asking how concerned respondents are about COVID-19 and its economic implications. We included these questions because we are only interested in measuring the feeling of anxiety (or concern) that is directly triggered by COVID-19 as one of the possible explanatory mechanisms for our findings; we are not interested in eliciting generic anxiety traits for our respondents. Beliefs about the ‘state of the world’ capture how serious respondents think COVID-19 is and how likely they think it is that others will follow government guidelines. The remaining variables, including those capturing different measures of social capital, patience, risk taking, altruism and sharing are elicited either via standard survey questions—similar to those used in the World Values Survey and other surveys—or by simple games (e.g., the dictator game). We include a detailed description of all these variables in S2 Appendix, with references to the survey instrument used to elicit them. Additionally, the complete survey instrument can be found in S3 Appendix.

### Empirical strategy

To estimate the treatment effect of the vaccine announcement we use ordinary least squares regressions. We estimate the following basic empirical model whereby *Y*_*i*_ is the vector of outcome variables, such as trust in government and elected politicians, *δ*_1_ is the treatment effect, *γ*_*i*_ is a vector of controls and *ϵ*_*i*_ the error term:

$$\begin{array}{c} Y_{i}=\beta_{0}+\delta_{1}vaccine+\gamma_{i}+\epsilon_{i} \end{array}$$ (1)

Our vector of controls includes gender, age, income, education and party affiliation. Party affiliation is of particular importance given that trust in government (and to some extent trust in elected politicians) is likely related to whether one supports the governing party or not. All our main specifications include these controls as well as robust standard errors clustered on the regional level (US states and UK NUTS-2 areas). We do so given the varying spread of COVID-19 as well as the different COVID-19-related measures in different US states and, to some extent, UK regions.

#### Robustness checks

To ensure the robustness of our main findings we conduct a series of robustness checks. First, we vary the clustering of our standard errors from state/region-level to hour-level (S5 Table). This is to account for the quickly moving news cycle in the hours and days after the vaccine announcement. We also run models with half day-clustered standard errors to account for possible heterogeneous time patterns of news consumption across different age and education groups (S6 Table). We then add state-/region-level fixed effects to test for the possibility that not just standard errors, but also coefficients may vary by state or region (S7 Table). We also rerun our main analysis with interactions rather than the ‘high exposure’ and ‘risk’ subgroups (S8 Table). Next, we rerun our analyses by news consumption as this potentially impacts the observed treatment effects of a news announcement (S9 Table). Finally, as our pre-treatment wave included an additional (separate) information experiment, we rerun our analyses using only data from respondents who did not receive the treatment, to ensure that the effects are not due to the information provided in the survey (S10 Table).

## Results

**Fig 2:**
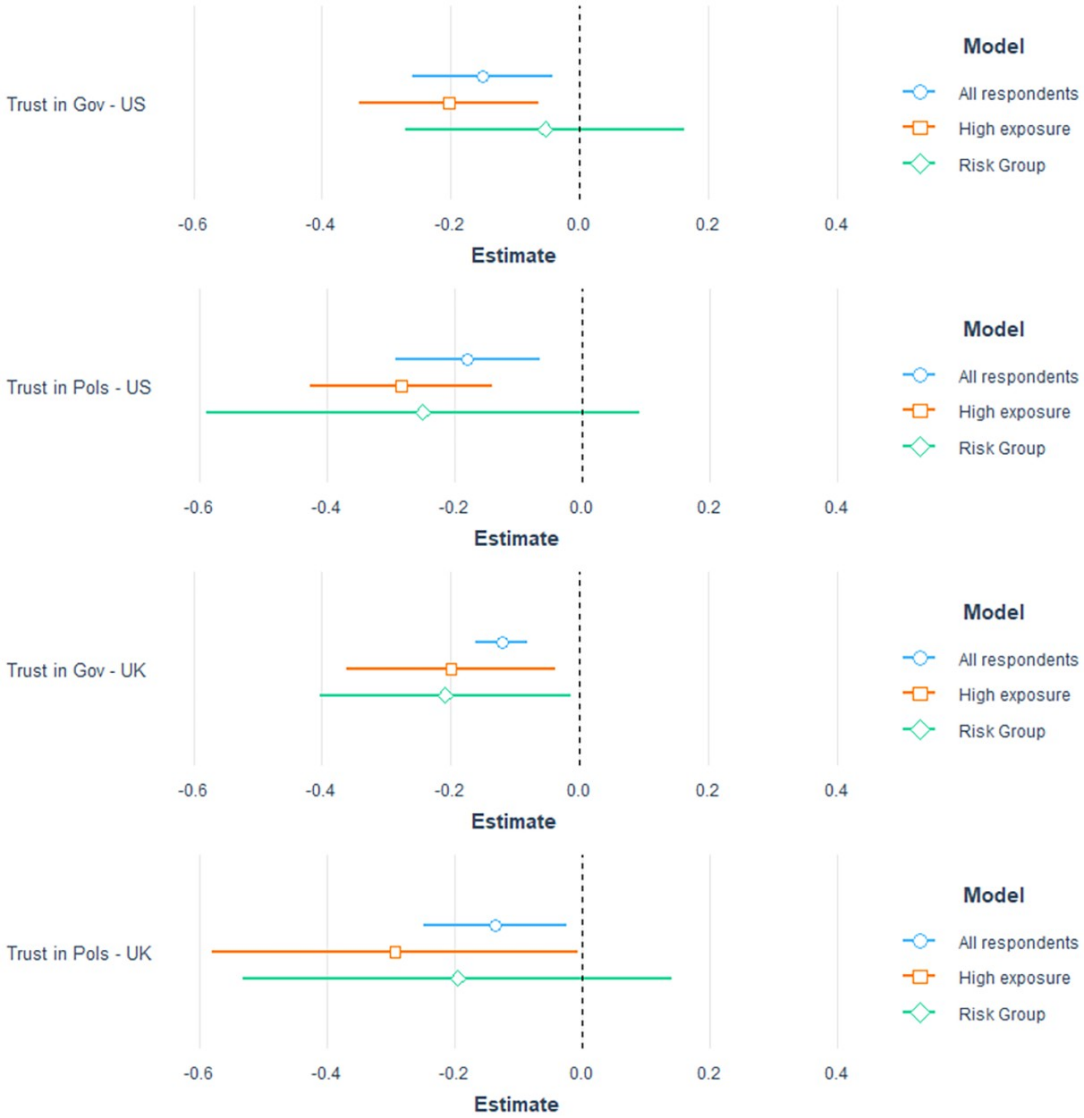
Treatment effects of vaccine announcement. Each estimate comes from an individual regression. Trust in government ranges from 1–4 and trust in politicians from 1–5, with higher values indicating a more positive assessment. Measures of anxiety range from 1 to 4 with higher values indicating more concern. Controls include gender, age, political affiliation, education and income. Standard errors are clustered on regions (UK) or states (US).

Fig 2 shows our main result: it reports the coefficient of the treatment variable explaining trust in government and trust in elected politicians, controlling also for the influence of age, gender, education, income and party support. We first look at the effect of the vaccine announcement on trust in government and elected politicians for all our respondents. We then zoom in on respondents who are highly exposed to catching the virus as they are required to commute for work on an above average number of days per week, and on respondents who are in the risk group, as defined by government guidelines in the US and UK, due to age or pre-existing medical conditions. These sub-groups may benefit especially from the vaccine.

Both in the US and the UK, we find that the vaccine announcement has a negative effect on trust in government and elected politicians. The coefficients for all respondents correspond to an average decrease in trust in government of about 28.6% in the US and 23.1% in the UK on a 4-point scale. This is the case, in both countries, when looking at all respondents, but becomes even stronger for respondents who are highly exposed in the US. Interestingly, those in the risk group—whom we would expect to be most sensitive to the vaccine announcement—show almost no reaction. These effect sizes are substantially larger in magnitude than, for example, the increase of 2.4–3.2% in trust in government on a similar 4-point scale reported at the start of the pandemic, when lockdowns were introduced [2].

Table 1 reports the coefficients for the effect of the vaccine treatment on a full set of belief, attitude, and behavior variables in our experiment. The models use standard survey weights to ensure the representativeness of our samples and allow us to draw inferences for the general US and UK populations. We also report p-values adjusted for multiple hypothesis testing, using the approach suggested by Anderson [36]. There are three outcome variables that enable us to check whether our respondents process the information included in the vaccine announcement in a rational way. First, we asked them to rate the seriousness of COVID-19 as compared with the seasonal flu. Their relative assessments in the both the UK and the US do not change, and since there is no reason to suppose that the vaccine—which affects catching and transmitting the virus—will alter how serious it is compared with seasonal flu, this is the (rational) response we would expect. Second, we asked our respondents a willingness to pay question for an intervention that reduces their chances of dying from COVID-19. This generally falls with the vaccine announcement, as should be expected since the reference likelihood of dying from COVID-19 that will be changed by the intervention falls after the announcement.

Finally, we have a consistency check on the beliefs of our respondents that comes from analyzing whether the vaccine announcement produces a change in their expectations about other people’s compliance with government guidelines that is consistent with what they say about their own behavior in the aggregate in this respect. On balance, the vaccine news-treated respondents neither expect others to change their compliance nor suggest that their own compliance will change as a result of the vaccine announcement.

Table 2 presents our main results with weights based on entropy balancing; this allows us to account for the minor imbalances detected (in S3 Table) and further corroborates the validity of our quasi-experimental design. Note that the results are substantively identical, and very similar in terms of the magnitude of the estimated effects, to those presented in Table 1. The other results are notable for the absence of any significant effect from the vaccine announcement. In particular, our indicators of social capital are rarely influenced by the news. Indeed, people’s levels of generalized trust—which is generally closely related to political trust, except that the object is not political—were not at all affected by the vaccine news. We also ran placebo checks testing the effect of the vaccine announcement on unrelated items included in our study and find no significant treatment effects (see S13 Table). S14 Table additionally reports balance tests for non-respondents of our main outcome variables, and shows that there are no significant differences between respondents and non-respondents. This all throws into sharp relief the consistent finding with respect to the significant effect of the vaccine announcement on trust in politicians.

Given the unexpected nature of our main finding, we rerun our analysis on data from the October/November 2020 Edelman Trust Barometer. The data allow us to test the effects of the vaccine announcement on trust in government and other institutions in the US (including the media, NGOs and business). We cannot run the same analysis for the UK as its respondents were interviewed before the vaccine announcement. The results of this analysis can be found in S15 Table. In line with the analysis of our own survey data, we find a significant negative effect of the vaccine announcement on trust in government. By contrast, we find no effect of the vaccine announcement on trust in other institutions. The Edelman data also enable us to test for overall time trends of trust in government as the fieldwork lasted from October 19 to November 18, 2020. Here, we find a weakly positive trend in governmental trust, suggesting that the effect of the vaccine announcement does not simply pick up on an overall trend in the outcome variable during the studied time period.

These results inevitably raise the following question. Given that the vaccine news did not concern the government, what might underpin this effect? Politicians—and governments in particular—did not have time to act on the vaccine announcement; they could only associate themselves with the news (see S1 Appendix). We suggest that the results are more likely to be driven by a mood effect on reasoning style, and we offer two possibilities: a form of motivated reasoning and a heuristic of relative comparison.

As regards the motivated reasoning mechanism, we conjecture that individuals’ self-esteem or self-image depends in part on their perceived self-efficacy [17] and that this, in turn, depends on an appropriate locus of control in their lives [37]. People need a modicum of uncertainty in their world and anxiety about their decision-making if they are to derive a sense of self-efficacy from those decisions. Too much uncertainty about the world and decisions are fraught with anxiety and difficult to make. Too little uncertainty and the task of decision-making loses any anxiety; decision-making becomes too mechanical to be a potent source of self-esteem. From this perspective, an exogenous shock to the uncertainty of the world and the anxiety that attends decision-making threatens people’s sense of self-efficacy and they will, where possible, adjust their beliefs so as to preserve the requisite sense of a locus of control in their lives. This is a form of motivated reasoning in the sense that beliefs are adjusted to support the motive of self-esteem. It could explain why trust in government soared with the onset of the pandemic [2, 3, 5]—a major exogenous injection of uncertainty and anxiety—and why trust in government then fell when the vaccine reduced that uncertainty and source of anxiety.

There is some evidence in support of this uncertainty/anxiety balancing movement of beliefs in government because there is little evidence that the vaccine announcement changes our respondents sense of anxiety. Indeed, the only two significant coefficients in Tables 1 and 2 with respect to our anxiety variables take opposite sign; the rest are not significantly different from zero. Likewise, there is no overall change in the perception of ‘luck versus effort’ in determining incomes with the vaccine announcement, suggesting little change in overall uncertainty. This is somewhat surprising given that our subjects show evidence of believing that the future has brightened with the vaccine announcement (e.g., through the willingness to pay responses), but it is less surprising if the adjustment of the belief in trust in government was designed to provide a compensating opposite change so as to stabilize levels of anxiety and perceived uncertainty at the requisite level for self-esteem.

**Fig 3:**
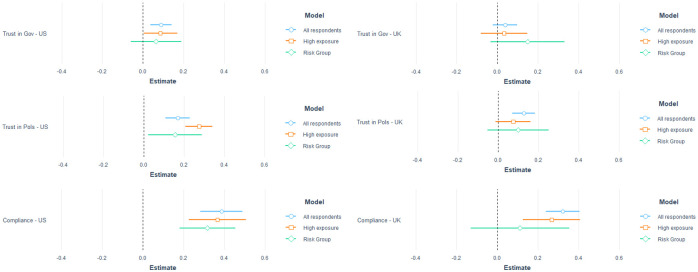
Effect of anxiety on trust in government and compliance with COVID-19 guidelines. Each estimate comes from an individual linear or logistic regression. Trust in government ranges from 1–4 and trust in politicians from 1–5, with higher values indicating a more positive assessment. Anxiety ranges from 1 to 4 with a higher value indicating more concern. Controls include gender, age, political affiliation, education and income. Standard errors are clustered on regions (UK) or states (US).

In addition, we find evidence for this potential mechanism when we examine individual differences in trust among our respondents. In Fig 3, we run individual-level regressions on trust, and include an additional explanatory variable of ‘concern for yourself and your family’ as a proxy for individual anxiety. We report the underlying regressions for in S11 Table. Again, we consider the same sub-groups and include the other controls. We also consider in the bottom two panels whether this anxiety variable helps predict individual compliance with government guidelines. We find that those who are most concerned about themselves and their family are also the ones who trust government the most. They are also the most likely to comply with government guidelines. Finally, S12 Table shows that when we interact our vaccine announcement treatment with our measure of concern, we find that those who are more concerned are more likely to significantly reduce their trust in government after the announcement.

The second possible reasoning mechanism is that of relative comparison. Though governments solely responded to the vaccine announcement by hailing it and, to some extent, associating it with their own past actions (see S1 Appendix), the news may have led citizens to put government competence in perspective, comparing it to the competence of the scientists who developed the vaccine. In such a comparison, governments will have appeared less potent and effective, which may, in turn, account for the decline in trust in government and politicians.

There is evidence to support this mechanism, too. While our survey did not inquire about citizens’ trust in scientists or other experts, Tables 1 and 2 show that evaluations of government competence were affected by the vaccine announcement in the same way that trust in government (and politicians) was. Moreover, S4 Table reports that in the US, where no changes to lockdown policies took place, the vaccine news negatively affected citizens’ evaluations of the introduction, easing and severity of lockdowns. By contrast, we find no effect of the vaccine news on lockdown evaluations in the UK, where a new (and long awaited) lockdown had been introduced in early November (before we conducted our survey). Here, any boost in government evaluations due to the lockdown may, in the vaccine news period, have been cancelled out by the countervailing effect of the comparison of government to scientists.

## Conclusion

Our results are surprising and carry implications for the evaluation of government efficacy and for the literature on the effect of good news. They are surprising in that they document a fundamental discord between citizens’ positive affect (and mood) following the vaccine announcement and their assessment of governments and their performance. To account for this, we suggest (a combination of) two possible mood effects on reasoning style. First, there is a form of motivated reasoning [29]. Our conjecture is that people’s beliefs are adjusted to support a requisite locus of control for decision-making to be a source of a sense of self-efficacy. At the onset, COVID-19 created a major new source of uncertainty and anxiety. People’s locus of (internal) control fell [37] and they needed to pin their hopes on something that could contain the new source of uncertainty and anxiety at levels required for self-efficacy. They adjusted their beliefs, where possible, accordingly. Governments are a possible agent for controlling COVID-19 uncertainty, and trust in government is a sufficiently subjective belief to admit adjustment. This may explain why trust in governments first rose. Yet, when the vaccine announcement was made, people had something else to pin their hopes on and so they re-adjusted their beliefs accordingly. As a result, trust in government fell. A similar mechanism has been found to be at work in task selection and mood-balancing. People who seek to optimize their positive affect choose to engage in mood-enhancing activities when they feel bad, and in unpleasant ones when they feel good [38]. Such a form of motivated reasoning aims at maximizing the sense of self-efficacy that people derive from their actions and emotions. Thus, it may well be that emotions—and the desire to balance mood and affect—shape behavior as well as beliefs and attitudes.

Second, there is the reasoning heuristic of relative comparison, which is similarly consistent with our findings (see Tables 1 and 2). Though the vaccine news did not affect governments, and politicians did not have the time to act on the news, (political) competency is not only evaluated in absolute terms, but also in relative terms. While the vaccine announcement shed a positive light on the competence of scientists, governments and elected politicians appeared relatively less potent and effective. This, in turn, might explain a decline in trust in politicians.

Irrespective of the exact interpretation, our results strongly suggest that factors other than governments’ own actions and performance can explain citizens’ trust in them, at least in the context of the pandemic. Moreover they point to a possible asymmetry between positive and negative news. The one is not the opposite of the other in its effect on behavior and beliefs, thus suggesting that the extant literature on negative news be complemented by one on good news.

## Supporting information

S1 Table Respondents per subgroup: United States. (ZIP)

S2 Table Respondents per subgroup: United Kingdom. (ZIP)

S3 Table Balance tests. (ZIP)

S4 Table Lockdown assessment in the US and the UK. (ZIP)

S5 Table Treatment effects of vaccine announcement with hour-robust standard errors. (ZIP)

S6 Table Treatment effects of vaccine announcement with half day-robust standard errors. (ZIP)

S7 Table Effect of vaccine announcement with state/region fixed effects. (ZIP)

S8 Table Treatment effects of vaccine announcement with interactions. (ZIP)

S9 Table Treatment effects by news consumption. (ZIP)

S10 Table Main treatment effects for control group only. (ZIP)

S11 Table Effect of concern and anxiety. (ZIP)

S12 Table Treatment interactions with concern. (ZIP)

S13 Table Placebo treatment effects. (ZIP)

S14 Table Balance test: Non-respondents of main variables. (ZIP)

S15 Table Treatment effects of vaccine announcement using Edelman data. (ZIP)

S1 Fig Media attention in the US and the UK. (ZIP)

S2 Fig US search interest in the presidential elections. (ZIP)

S1 Appendix Coincidental and collateral events. (ZIP)

S2 Appendix Description of main variables. (ZIP)

S3 Appendix Survey instrument. (ZIP)
