# Supplementary material for: Good news reduces trust in government and its efficacy: The case of the Pfizer/BioNTech vaccine announcement
Source: PLoS One. 2021 Dec 9;16(12):e0260216. doi: 10.1371/journal.pone.0260216 (PMC8659308; doi:10.1371/journal.pone.0260216)
Supplement: S1 Table — (ZIP) [file pone.0260216.s001.zip › s1_table.pdf]

S1 Table. Respondents per subgroup: United States

	Work status							
	Employed (also incl. unemployed 65+)				Unemployed			
	**Regions/divisions**				**Regions/divisions**			
	Northeast	Midwest	South	West	Northeast	Midwest	South	West
Pre-treatment								
**Female**								
**Age**								
18-24	6	11	14	10	4	4	9	6
25-34	7	11	18	15	3	2	7	6
35-44	9	4	14	11	3	1	8	3
45-54	7	8	9	10	1	2	5	2
55-64	4	7	9	3	3	3	3	4
65+	9	12	26	14				
**Male**								
**Age**								
18-24	6	9	16	10	5	4	10	6
25-34	10	12	22	20	3	0	4	3
35-44	9	10	18	13	1	1	2	2
45-54	6	11	13	14	0	0	5	3
55-64	9	9	12	12	4	0	6	3
65+	5	7	16	8				
Post-treatment								
**Female**								
**Age**								
18-24	4	6	9	6	3	3	7	4
25-34	8	10	17	11	3	3	7	5
35-44	7	9	16	10	3	3	6	4
45-54	8	9	16	10	3	3	7	4
55-64	7	8	13	8	4	5	11	6
65+	15	17	30	17				
**Male**								
**Age**								
18-24	4	6	9	6	3	3	7	4
25-34	9	11	19	14	2	2	4	3
35-44	8	10	18	13	1	1	3	2
45-54	8	10	18	11	2	2	4	2
55-64	7	9	14	9	3	3	7	4
65+	12	14	24	15				
