# Supplementary material for: Good news reduces trust in government and its efficacy: The case of the Pfizer/BioNTech vaccine announcement
Source: PLoS One. 2021 Dec 9;16(12):e0260216. doi: 10.1371/journal.pone.0260216 (PMC8659308; doi:10.1371/journal.pone.0260216)
Supplement: S2 Table — (ZIP) [file pone.0260216.s002.zip › s2_table.pdf]

S2 Table. Respondents by subgroup: United Kingdom

	**Work status**								
	Employed (also incl. unemployed 65+)					Unemployed			
	**Regions/divisions**					**Regions/divisions**			
	North	Midlands	South	Wales	Scotland	North	Midlands	South	Wales
Pre-treatment									
**Female**									
**Age**									
16-24	9	10	14	2	4	7	7	9	1
25-34	9	11	15	2	5	3	4	4	1
35-49	10	15	16	2	7	2	6	7	1
50-64	3	5	5	1	4	3	2	3	1
65+	11	9	6	3	5				
**Male**									
**Age**									
16-24	8	9	12	2	7	8	8	13	3
25-34	10	13	20	2	4	3	2	3	0
35-49	10	9	16	2	6	2	3	1	0
50-64	5	5	7	1	3	3	1	1	0
65+	13	19	21	4	7				
Post-treatment									
**Female**									
**Age**									
16-24	6	7	9	1	4	6	6	9	1
25-34	10	10	17	2	5	4	4	6	1
35-49	17	19	26	3	8	5	5	8	1
50-64	11	13	17	2	7	8	8	10	2
65+	20	21	27	4	7				
**Male**									
**Age**									
16-24	6	7	9	1	4	7	6	9	1
25-34	11	12	20	2	5	2	2	3	1
35-49	18	21	29	4	8	4	3	4	1
50-64	18	21	29	4	8	4	3	4	1
65+	15	17	21	4	5				
