# Supplementary material for: Good news reduces trust in government and its efficacy: The case of the Pfizer/BioNTech vaccine announcement
Source: PLoS One. 2021 Dec 9;16(12):e0260216. doi: 10.1371/journal.pone.0260216 (PMC8659308; doi:10.1371/journal.pone.0260216)
Supplement: S3 Table — (ZIP) [file pone.0260216.s003.zip › s3_table.pdf]

S3 Table. Balance tests

	United States			United Kingdom		
	Control	Treatment	t-statistic	Control	Treatment	t-statistic
Gender	0.49	0.52	-1.13	0.48	0.52	-1.12
	(0.50)	(0.50)	(1,344)	(0.50)	(0.50)	(1,216)
Age	40.62	46.46	-6.47***	39.90	44.93	-5.06***
	(16.44)	(16.94)	(1,372)	(17.84)	(16.87)	(1,232)
Education	3.71	3.75	-1.57	3.75	3.72	0.82
	(0.48)	(0.44)	(1,331)	(0.45)	(0.47)	(1,198)
Income	5.89	5.59	2.01**	4.25	3.78	3.67***
	(2.53)	(2.62)	(1,259)	(2.13)	(2.08)	(1,117)
Not in work	0.14	0.14	-0.00	0.13	0.22	-4.38***
	(0.35)	(0.35)	(1,379)	(0.33)	(0.42)	(1,234)

Notes: Table reports the mean values for the pre- and post-announcement samples. 'Gender' takes the value of 1 for female; 'age' runs from 18 to 89 (continuous); 'education' ranges from 1 to 4; 'income' ranges from 1 to 10; 'not in work' takes the value of 1 when people are not in paid work or on furlough. Asterisks indicate significant differences in mean values between samples from a Wald test of significance (with degrees of freedom in parentheses). Standard deviations are below the means, in parentheses. *** $p<0.01$, ** $p<0.05$, * $p<0.1$.

Though our respondents were not randomly assigned to the control and treatment groups, we consider the assignment of respondents to the groups as independent from the occurrence of the announcement and 'as good as random', especially as we newly recruited respondents based on the same set of quotas. We nonetheless performed difference of means tests on the demographic composition of the two groups. S3 Table shows that some of the covariates are imbalanced between the groups. We therefore present our main models in two ways: using sample weights (Table 1) and using entropy balance weights (Table 2) [34]. Moreover, we control for gender, age, education and income, as well as for political affiliation as this is a key factor explaining evaluations of the (current) government.
