# Supplementary material for: Good news reduces trust in government and its efficacy: The case of the Pfizer/BioNTech vaccine announcement
Source: PLoS One. 2021 Dec 9;16(12):e0260216. doi: 10.1371/journal.pone.0260216 (PMC8659308; doi:10.1371/journal.pone.0260216)
Supplement: S4 Table — (ZIP) [file pone.0260216.s004.zip › s4_table.pdf]

S4 Table. Lockdown assessment in the US and the UK

	United States			United Kingdom		
	All respondents	Highly exposed	Risk group	All respondents	Highly exposed	Risk group
Introduction of lockdowns	-0.114*	-0.005	-0.114	-0.048	-0.036	-0.270***
	(0.057)	(0.091)	(0.114)	(0.054)	(0.060)	(0.063)
Easing of lockdowns	-0.164***	-0.108	-0.206**	-0.060	0.019	-0.012
	(0.034)	(0.074)	(0.093)	(0.081)	(0.100)	(0.096)
Severity of lockdowns	-0.109*	-0.062	-0.075	-0.042	0.040	-0.077
	(0.054)	(0.089)	(0.132)	(0.084)	(0.094)	(0.155)
Observations	1,170	517	404	927	352	195

Notes: Each estimate comes from an individual linear regression. Variables ranges from 1 to 3 with a higher value indicating a more positive assessment of the introduction, easing and severity of lockdown measures. Controls include gender, age, political affiliation, education and income. State- and region-clustered standard errors are in parenthesis. *** p<0.01, ** p<0.05 , * p<0.1.
