# Supplementary material for: Good news reduces trust in government and its efficacy: The case of the Pfizer/BioNTech vaccine announcement
Source: PLoS One. 2021 Dec 9;16(12):e0260216. doi: 10.1371/journal.pone.0260216 (PMC8659308; doi:10.1371/journal.pone.0260216)
Supplement: S6 Table — (ZIP) [file pone.0260216.s006.zip › s6_table.pdf]

S6 Table. Treatment effects of vaccine announcement with half day-robust standard errors

	United States			United Kingdom		
	All respondents	Highly exposed	Risk group	All respondents	Highly exposed	Risk group
**Government assessment**						
Trust in government	-0.149**	-0.205**	-0.050	-0.117***	-0.199***	-0.204**
	(0.047)	(0.051)	(0.133)	(0.003)	(0.017)	(0.038)
Trust in politicians	-0.183**	-0.297**	-0.239*	-0.134**	-0.290***	-0.193**
	(0.047)	(0.095)	(0.086)	(0.027)	(0.025)	(0.045)
Government competency	-0.164***	-0.137	-0.157	-0.163**	-0.217***	-0.280***
	(0.020)	(0.075)	(0.111)	(0.032)	(0.009)	(0.045)
**Measures of anxiety**						
Concern	0.118	-0.058	0.100*	0.038***	0.044	-0.106
	(0.071)	(0.025)	(0.039)	(0.006)	(0.044)	(0.048)
Economic concern	0.062***	0.038	-0.058	-0.091***	-0.112*	-0.227**
	(0.013)	(0.065)	(0.095)	(0.012)	(0.044)	(0.039)
**Beliefs about the world**						
Seriousness	0.041	-0.015	0.064	-0.005	-0.039	-0.022
	(0.027)	(0.029)	(0.057)	(0.007)	(0.025)	(0.038)
Others follow guidelines	-0.122*	-0.198	-0.125	0.001	-0.135**	0.077
	(0.054)	(0.123)	(0.138)	(0.022)	(0.027)	(0.054)
Luck vs. effort	-0.022	0.005	0.238	-0.210**	-0.423***	-1.177**
	(0.131)	(0.226)	(0.429)	(0.038)	(0.023)	(0.260)
**Elicited behaviors**						
Willingness to pay	1.545	-20.724**	8.369	-10.864***	-8.021*	-25.325**
	(3.862)	(5.469)	(10.641)	(1.323)	(2.541)	(7.274)
Willingness to comply	-0.037	-0.133*	0.156	0.004	0.092***	0.067
	(0.038)	(0.050)	(0.080)	(0.011)	(0.008)	(0.048)
**Social capital**						
Patience	-0.057	0.068	0.301	-0.299**	-0.180*	-0.718**
	(0.054)	(0.273)	(0.296)	(0.090)	(0.073)	(0.136)
Generalized trust	0.042	0.054*	0.032	-0.015**	-0.072**	0.074***
	(0.026)	(0.021)	(0.020)	(0.004)	(0.013)	(0.012)
Risk taking	-0.250	-0.197*	-0.440	-0.063**	0.247***	-0.030
	(0.123)	(0.081)	(0.366)	(0.019)	(0.026)	(0.109)
Dictator game sharing	0.034	0.300**	0.026	-0.031	-0.012	-0.400
	(0.078)	(0.107)	(0.071)	(0.073)	(0.187)	(0.199)
Altruism	1.375	-28.133	22.845	-4.102	13.697*	-13.951
	(3.757)	(16.369)	(23.459)	(2.230)	(5.189)	(10.580)
Observations	1,381	605	448	1,236	457	234

Notes: Each estimate comes from an individual linear regression. Trust in government ranges from 1-4, trust in politicians and government competency from 1-5 with higher values indicating a more positive assessment. Measures of anxiety range from 1 to 4 with higher values indicating more concern. Seriousness (1-4) captures the perceived seriousness of COVID-19 compared to the flu. Others follow guidelines (1-5) captures the perceived likelihood that others comply with government guidelines. Luck vs. Effort (0-10) indicates whether income differences are perceived to result from luck (0) or from effort (10). Willingness to pay ranges from $/£0 to £200/$260 capturing the amount respondent i is willing to pay for a treatment to reduce own mortality from COVID-19. Willingness to comply (1-4) captures the self-reported likelihood to comply with guidelines. For all social capital variables, higher values indicate more patience (0-10), trust (0-1), willingness to take risks (0-10), dictator game sharing (0-10) and altruism (0-1000). Controls include gender, age, political affiliation, education and income. Half day clustered standard errors are in parenthesis. *** p<0.01, ** p<0.05 , * p<0.1.

S6 Table reports our main results with half day-clustered standard errors, to take into account the possibility that time patterns of news consumption may vary across groups. For each survey day, we distinguished between day

time (6am to 6pm) and evening/night time (6pm to 6am), and created a variable that captured, for each time zone in our data, the half day in which the survey was filled out.

Our main results remain the same as with region-clustered standard errors, with trust in government and trust in elected politicians being significantly lower post-vaccine announcement than prior to the announcement. Furthermore, like in the main analyses, we find some evidence that government competency is assessed more negatively, especially in the UK, and especially for those who are exposed and in the risk group. Apart from this significant treatment effect on government assessment, we do not find consistent effects of the vaccine news on the other categories of variables.
