# Supplementary material for: Good news reduces trust in government and its efficacy: The case of the Pfizer/BioNTech vaccine announcement
Source: PLoS One. 2021 Dec 9;16(12):e0260216. doi: 10.1371/journal.pone.0260216 (PMC8659308; doi:10.1371/journal.pone.0260216)
Supplement: S7 Table — (ZIP) [file pone.0260216.s007.zip › s7_table.pdf]

S7 Table. Effect of vaccine announcement with state/region fixed effects

	United States			United Kingdom		
	All respondents	Highly exposed	Risk group	All respondents	Highly exposed	Risk group
**Government assessment**						
Trust in government	-0.152***	-0.219***	-0.010	-0.123***	-0.205***	-0.192***
	(0.045)	(0.074)	(0.083)	(0.033)	(0.052)	(0.064)
Trust in politicians	-0.197***	-0.262***	-0.290**	-0.139*	-0.303***	-0.142
	(0.060)	(0.081)	(0.140)	(0.069)	(0.041)	(0.136)
Government competency	-0.149**	-0.118	-0.145	-0.155**	-0.216***	-0.198
	(0.069)	(0.076)	(0.140)	(0.070)	(0.069)	(0.142)
**Measures of anxiety**						
Concern	0.129**	-0.040	0.079	0.037	0.056	-0.151
	(0.052)	(0.092)	(0.098)	(0.067)	(0.074)	(0.104)
Economic concern	0.082**	0.046	-0.026	-0.089**	-0.118	-0.213
	(0.032)	(0.036)	(0.053)	(0.041)	(0.072)	(0.130)
**Beliefs about the world**						
Seriousness	0.032	-0.027	0.030	-0.007	-0.039	-0.030
	(0.037)	(0.065)	(0.065)	(0.053)	(0.055)	(0.058)
Others follow guidelines	-0.153*	-0.180**	-0.178	-0.005	-0.156	0.112
	(0.088)	(0.068)	(0.127)	(0.062)	(0.120)	(0.157)
Luck vs. effort	-0.054	-0.090	0.058	-0.181	-0.403	-1.182**
	(0.138)	(0.278)	(0.514)	(0.177)	(0.374)	(0.483)
**Elicited behaviors**						
Willingness to pay	-1.483	-19.785***	5.628	-11.316***	-9.015**	-28.424***
	(5.146)	(6.098)	(9.701)	(3.442)	(4.232)	(9.453)
Willingness to comply	-0.023	-0.072	0.164	-0.003	0.083	0.084
	(0.054)	(0.079)	(0.119)	(0.051)	(0.075)	(0.103)
**Social capital**						
Patience	-0.157	0.009	0.245	-0.299*	-0.100	-0.768**
	(0.169)	(0.300)	(0.369)	(0.159)	(0.194)	(0.358)
Generalized trust	0.045	0.050	0.043	-0.017	-0.085**	0.093
	(0.030)	(0.034)	(0.083)	(0.021)	(0.033)	(0.060)
Risk taking	-0.219*	-0.169	-0.429*	-0.055	0.290*	-0.189
	(0.092)	(0.176)	(0.220)	(0.111)	(0.145)	(0.335)
Dictator game sharing	-0.029	0.325*	-0.094	-0.027	-0.034	-0.385
	(0.072)	(0.175)	(0.233)	(0.103)	(0.254)	(0.356)
Altruism	-10.144	-21.674*	6.875	-3.540	11.749	-15.809
	(13.608)	(11.021)	(26.012)	(8.655)	(12.561)	(14.993)
Observations	1,381	605	448	1,236	457	234

Notes: Each estimate comes from an individual linear regression. Trust in government ranges from 1-4, trust in politicians and government competency from 1-5 with higher values indicating a more positive assessment. Measures of anxiety range from 1 to 4 with higher values indicating more concern. Seriousness (1-4) captures the perceived seriousness of COVID-19 compared to the flu. Others follow guidelines (1-5) captures the perceived likelihood that others comply with government guidelines. Luck vs. Effort (0-10) indicates whether income differences are perceived to result from luck (0) or from effort (10). Willingness to pay ranges from $/£0 to £200/$260 capturing the amount respondent i is willing to pay for a treatment to reduce own mortality from COVID-19. Willingness to comply (1-4) captures the self-reported likelihood to comply with guidelines. For all social capital variables, higher values indicate more patience (0-10), trust (0-1), willingness to take risks (0-10), dictator game sharing (0-10) and altruism (0-1000). Controls include gender, age, political affiliation, education and income. Hour-clustered standard errors are in parenthesis. *** p<0.01, ** p<0.05 , * p<0.1.

S7 Table reports our main results with hour-clustered standard errors and additional region-/state-level fixed effects. We again look first at the effects of the vaccine news for all respondents and then at those who are highly exposed or self-identity as in the risk group. We again find strong evidence for our main finding. Trust in government and

trust in elected politicians are significantly and negatively affected by the vaccine announcement. There is again also some evidence for a more negative assessment of government competency in both countries. Apart from our main treatment effect, we also find a significant reduction in the willingness to pay of respondents in the UK and highly exposed respondents in the US.
