# Supplementary material for: Good news reduces trust in government and its efficacy: The case of the Pfizer/BioNTech vaccine announcement
Source: PLoS One. 2021 Dec 9;16(12):e0260216. doi: 10.1371/journal.pone.0260216 (PMC8659308; doi:10.1371/journal.pone.0260216)
Supplement: S8 Table — (ZIP) [file pone.0260216.s008.zip › s8_table.pdf]

S8 Table. Treatment effects of vaccine announcement with interactions

	United States		United Kingdom	
	Highly exposed	Risk group	Highly exposed	Risk group
**Government assessment**				
Trust in government	-0.112*	0.020	-0.056	-0.007
	(0.062)	(0.063)	(0.087)	(0.052)
Trust in politicians	-0.213***	-0.016	-0.190*	-0.065
	(0.068)	(0.077)	(0.101)	(0.075)
Government competency	0.072	-0.096	-0.142	0.015
	(0.096)	(0.095)	(0.117)	(0.103)
**Measures of anxiety**				
Concern	-0.126**	0.333***	0.047	0.213**
	(0.061)	(0.100)	(0.072)	(0.079)
Economic concern	0.008	0.070	-0.119***	-0.179***
	(0.085)	(0.053)	(0.027)	(0.045)
**Beliefs about the world**				
Seriousness	-0.071	0.134**	0.022	0.170***
	(0.066)	(0.052)	(0.041)	(0.037)
Others follow guidelines	-0.121	-0.063	-0.135	-0.112
	(0.080)	(0.085)	(0.167)	(0.090)
Luck vs. effort	0.001	0.203	-0.257	-0.490**
	(0.250)	(0.200)	(0.215)	(0.194)
**Elicited behaviors**				
Willingness to pay	-19.430***	10.823*	-6.342	-2.782
	(6.351)	(6.292)	(10.177)	(7.091)
Willingness to comply	-0.129	0.203***	0.115	0.073
	(0.084)	(0.074)	(0.078)	(0.078)
**Social capital**				
Patience	0.206	-0.147	-0.055	-0.479**
	(0.216)	(0.187)	(0.246)	(0.188)
Generalized trust	0.025	0.032	-0.081	-0.028
	(0.042)	(0.045)	(0.057)	(0.043)
Risk taking	0.058	-0.483**	0.085	-0.487**
	(0.189)	(0.214)	(0.193)	(0.183)
Dictator game sharing	0.157	0.160	-0.046	0.061
	(0.175)	(0.167)	(0.270)	(0.239)
Altruism	-35.403**	27.676	13.539	-8.972
	(14.699)	(19.329)	(17.045)	(13.938)
Observations	1,174	1,129	916	881

Notes: Each estimate comes from an individual linear regression. Trust in government ranges from 1-4, trust in politicians and government competency from 1-5 with higher values indicating a more positive assessment. Measures of anxiety range from 1 to 4 with higher values indicating more concern. Seriousness (1-4) captures the perceived seriousness of COVID-19 compared to the flu. Others follow guidelines (1-5) captures the perceived likelihood that others comply with government guidelines. Luck vs. Effort (0-10) indicates whether income differences are perceived to result from luck (0) or from effort (10). Willingness to pay ranges from $/£0 to £200/$260 capturing the amount respondent i is willing to pay for a treatment to reduce own mortality from COVID-19. Willingness to comply (1-4) captures the self-reported likelihood to comply with guidelines. For all social capital variables, higher values indicate more patience (0-10), trust (0-1), willingness to take risks (0-10), dictator game sharing (0-10) and altruism (0-1000). Controls include gender, age, political affiliation, education and income. Hour-clustered standard errors are in parenthesis. *** p<0.01, ** p<0.05, * p<0.1.

S8 Table reports our main results with interactions, rather than subgroups. Each coefficient reports the effect of the vaccine announcement interacted with being in the specified subgroup.
