# Supplementary material for: Good news reduces trust in government and its efficacy: The case of the Pfizer/BioNTech vaccine announcement
Source: PLoS One. 2021 Dec 9;16(12):e0260216. doi: 10.1371/journal.pone.0260216 (PMC8659308; doi:10.1371/journal.pone.0260216)
Supplement: S9 Table — (ZIP) [file pone.0260216.s009.zip › s9_table.pdf]

S9 Table. Treatment effects by news consumption

	United States		United Kingdom	
	High	Low	High	Low
**Government assessment**				
Trust in government	-0.217***	-0.080	-0.066	-0.185***
	(0.077)	(0.068)	(0.052)	(0.046)
Trust in politicians	-0.310***	0.045	-0.110	-0.125**
	(0.089)	(0.072)	(0.109)	(0.047)
Government competency	-0.178*	-0.202**	-0.101	-0.245***
	(0.102)	(0.093)	(0.127)	(0.069)
**Measures of anxiety**				
Concern	0.200**	0.120	0.131	-0.004
	(0.092)	(0.081)	(0.117)	(0.054)
Economic concern	0.151**	0.047	-0.111	-0.070
	(0.064)	(0.076)	(0.075)	(0.046)
**Beliefs about the world**				
Seriousness	0.061	0.075	-0.010	0.027
	(0.045)	(0.058)	(0.060)	(0.062)
Others follow guidelines	-0.171	-0.053	-0.022	0.008
	(0.125)	(0.082)	(0.117)	(0.111)
Luck vs. effort	0.076	-0.376	-0.268	-0.135
	(0.305)	(0.309)	(0.371)	(0.281)
**Elicited behaviors**				
Willingness to pay	3.097	5.589	-5.256	-13.963
	(8.607)	(7.504)	(3.692)	(8.552)
Willingness to comply	-0.026	0.008	0.022	-0.012
	(0.069)	(0.077)	(0.085)	(0.072)
**Social capital**				
Patience	-0.024	-0.087	-0.444	-0.115
	(0.208)	(0.232)	(0.250)	(0.279)
Generalized trust	0.026	0.065	-0.107**	0.052
	(0.044)	(0.048)	(0.044)	(0.037)
Risk taking	-0.439*	-0.015	-0.021	-0.054
	(0.234)	(0.181)	(0.306)	(0.268)
Dictator game sharing	0.098	0.053	0.091	-0.164
	(0.224)	(0.189)	(0.168)	(0.161)
Altruism	-7.852	8.577	-17.419	5.785
	(18.553)	(17.241)	(16.367)	(13.200)
Observations	692	636	500	695

Notes: Each estimate comes from an individual linear regression. Trust in government ranges from 1-4, trust in politicians and government competency from 1-5 with higher values indicating a more positive assessment. Measures of anxiety range from 1 to 4 with higher values indicating more concern. Seriousness (1-4) captures the perceived seriousness of COVID-19 compared to the flu. Others follow guidelines (1-5) captures the perceived likelihood that others comply with government guidelines. Luck vs. Effort (0-10) indicates whether income differences are perceived to result from luck (0) or from effort (10). Willingness to pay ranges from $/£0 to £200/$260 capturing the amount respondent i is willing to pay for a treatment to reduce own mortality from COVID-19. Willingness to comply (1-4) captures the self-reported likelihood to comply with guidelines. For all social capital variables, higher values indicate more patience (0-10), trust (0-1), willingness to take risks (0-10), dictator game sharing (0-10) and altruism (0-1000). Controls include gender, age, political affiliation, education and income. State- and region-clustered standard errors are in parenthesis. *** $p<0.01$, ** $p<0.05$, * $p<0.1$.

S9 Table reports our main results by news consumption. For this specification, we divide our respondents into those who consume a large amount of news and those who do not. To do so, we use question D15 in the UK and D16 in the US. Respondents who indicated that they consume more than 1hr of news per day are classified as "high news consumers", everyone else is classified as a "low news consumer". As the table reports, our main result is primarily driven by high news consumers in the US and by low news consumers in the UK. This is somewhat surprising. Given that we control for party affiliation in all our specifications this cannot be related to respondents with either political affiliation consuming more or less news.
