# Supplementary material for: Good news reduces trust in government and its efficacy: The case of the Pfizer/BioNTech vaccine announcement
Source: PLoS One. 2021 Dec 9;16(12):e0260216. doi: 10.1371/journal.pone.0260216 (PMC8659308; doi:10.1371/journal.pone.0260216)
Supplement: S10 Table — (ZIP) [file pone.0260216.s010.zip › s10_table.pdf]

S10 Table. Main treatment effects for control group only

	United States			United Kingdom		
	All respondents	Highly exposed	Risk group	All respondents	Highly exposed	Risk group
Trust in government	-0.121	-0.275***	0.020	-0.146***	-0.316***	-0.034
	0.083)	0.084)	(0.146)	(0.043)	(0.085)	(0.135)
Trust in politicians	-0.233**	-0.360***	-0.404*	-0.177	-0.364**	0.153
	(0.099)	(0.097)	(0.239)	(0.102)	(0.121)	(0.227)
Government competency	-0.065	-0.092	-0.168	-0.217**	-0.374***	-0.104
	(0.078)	(0.178)	(0.149)	(0.091)	(0.098)	(0.156)
Observations	940	285	329	897	253	182

Notes: Each estimate comes from an individual linear regression. Trust in government ranges from 1-4, trust in politicians and government competency from 1-5 with higher values indicating a more positive assessment. Controls include gender, age, political affiliation, education and income. State- and region-clustered standard errors are in parenthesis. *** p<0.01, ** p<0.05 , * p<0.1.

In our pre-treatment wave, respondents were divided into control and treatment groups as part of an information experiment. The treatment groups received information about the number of expected deaths in their respective country and the expected economic costs of the crisis in the year 2020. To ensure that our observed treatment effects from the vaccine announcement are not due to this information treatment, which was not repeated in the post-treatment wave, we check the robustness of our main effects for the control group of our pre-treatment wave only. S10 Table reports the results. While not all coefficients remain statistically significant, which is most likely due to the significantly smaller sample size, our main results hold.
