# Supplementary material for: Good news reduces trust in government and its efficacy: The case of the Pfizer/BioNTech vaccine announcement
Source: PLoS One. 2021 Dec 9;16(12):e0260216. doi: 10.1371/journal.pone.0260216 (PMC8659308; doi:10.1371/journal.pone.0260216)
Supplement: S11 Table — (ZIP) [file pone.0260216.s011.zip › s11_table.pdf]

S11 Table. Effect of concern and anxiety

	United States			United Kingdom		
	All respondents	Highly exposed	Risk group	All respondents	Highly exposed	Risk group
Trust in government	0.089***	0.085**	0.064	0.038	0.035	0.150*
	(0.025)	(0.038)	(0.058)	(0.026)	(0.048)	(0.076)
Trust in politicians	0.169***	0.273***	0.156**	0.128***	0.076*	0.100
	(0.029)	(0.032)	(0.064)	(0.024)	(0.036)	(0.060)
Compliance	0.386***	0.366***	0.316***	0.321***	0.268***	0.112
	(0.049)	(0.066)	(0.065)	(0.036)	(0.059)	(0.099)
Observations	1,381	605	448	1,236	457	234

Notes: Each estimate comes from an individual linear regression. Trust in government ranges from 1-4, trust in politicians and government competency from 1-5 with higher values indicating a more positive assessment. Anxiety ranges from 1 to 4 with a higher value indicating more concern. Controls include gender, age, political affiliation, education and income. State- and region-clustered standard errors are in parenthesis. *** $p<0.01$, ** $p<0.05$, * $p<0.1$.

S11 Table reports the regressions underlying Fig 3 in the main text. As discussed there, we find that those who are most concerned about themselves and their family, with the highest level of anxiety or concern due to COVID-19, are also those who trust government, in particular elected politicians, the most. They are also the most likely to comply with government guidelines.
