# Supplementary material for: Good news reduces trust in government and its efficacy: The case of the Pfizer/BioNTech vaccine announcement
Source: PLoS One. 2021 Dec 9;16(12):e0260216. doi: 10.1371/journal.pone.0260216 (PMC8659308; doi:10.1371/journal.pone.0260216)
Supplement: S12 Table — (ZIP) [file pone.0260216.s012.zip › s12_table.pdf]

S12 Table. Treatment interactions with concern

	United States			United Kingdom		
	All respondents	Highly exposed	Risk group	All respondents	Highly exposed	Risk group
Trust in government	-0.043	-0.168**	-0.061	-0.076*	-0.142	0.140
	(0.045)	(0.082)	(0.070)	(0.041)	(0.099)	(0.127)
Trust in politicians	-0.057	-0.229**	-0.151	-0.091	-0.259	-0.089
	(0.046)	(0.092)	(0.092)	(0.060)	(0.168)	(0.164)
Gov competency	-0.306***	-0.316	-0.235**	-0.187**	-0.200	-0.071
	(0.063)	(0.190)	(0.093)	(0.074)	(0.134)	(0.139)
Observations	1,172	518	404	915	350	194

Notes: Each estimate comes from individual linear regressions. Trust in government ranges from 1-4, trust in politicians and government competency from 1-5 with higher values indicating a more positive assessment. Concern is measured as a binary variable with 1 indicating a "very concerned" response and 0 otherwise. Controls include gender, age, political affiliation, education and income. State- and region-clustered standard errors are in parenthesis. *** p<0.01, ** p<0.05 , * p<0.1.

S12 Table reports a further test of our proposed mechanism. Each coefficient reports the interaction term between our treatment (post-vaccine announcement) and our measure of concern due to Covid-19. While not all models reach conventional levels of statistical significance as the sample size is somewhat reduced, the overall pattern illustrates clearly that the observed treatment effect is driven by those respondents with high levels of concern. This is especially the case for those respondents who are highly exposed.
