# Supplementary material for: Good news reduces trust in government and its efficacy: The case of the Pfizer/BioNTech vaccine announcement
Source: PLoS One. 2021 Dec 9;16(12):e0260216. doi: 10.1371/journal.pone.0260216 (PMC8659308; doi:10.1371/journal.pone.0260216)
Supplement: S13 Table — (ZIP) [file pone.0260216.s013.zip › s13_table.pdf]

S13 Table. Placebo treatment effects

	United States			United Kingdom		
	All respondents	Highly exposed	Risk group	All respondents	Highly exposed	Risk group
Climate Change	0.013	0.014	0.061	0.046	0.108*	0.037
	(0.019)	(0.032)	(0.043)	(0.026)	(0.055)	(0.074)
UBI	0.026	-0.197	0.004	0.089	-0.100	0.210
	(0.079)	(0.130)	(0.175)	(0.067)	(0.143)	(0.225)
Redistribution	0.053	-0.249	0.350	-0.270*	-0.148	-0.777
	(0.176)	(0.222)	(0.415)	(0.133)	(0.142)	(0.477)
Observations	1,126	501	394	854	324	180

Notes: Each estimate comes from individual linear regressions. Climate change is a binary variable with 1 indicating agreement with the statement "The government could do more to tackle climate change". UBI ranges from 1 to 5 with 5 indicating strong support for the government introducing a Universal Basic Income. Redistribution ranges from 0 to 10 with 10 indicating the least support for income redistribution by the government. Controls include gender, age, political affiliation, education and income. State- and region-clustered standard errors are in parenthesis. *** p<0.01, ** p<0.05 , * p<0.1.

S13 Table reports three placebo checks testing the effects of our treatment, the vaccine announcement, on questions unrelated to our main variables of interest. We find no evidence for treatment effects on the reported variables. The vaccine announcement did not change support for climate change policies, universal basic income or general income redistribution in either the US or UK.
