# Supplementary material for: Good news reduces trust in government and its efficacy: The case of the Pfizer/BioNTech vaccine announcement
Source: PLoS One. 2021 Dec 9;16(12):e0260216. doi: 10.1371/journal.pone.0260216 (PMC8659308; doi:10.1371/journal.pone.0260216)
Supplement: S14 Table — (ZIP) [file pone.0260216.s014.zip › s14_table.pdf]

S14 Table. Balance test: Non-respondents of main variables

	United States			United Kingdom		
	Respondent	Non-respondent	t-statistic	Respondent	Non-respondent	t-statistic
Trust in government						
Gender	0.50	0.57	0.84	0.50	0.63	1.85*
	(0.50)	(0.50)	(1,344)	(0.50)	(0.49)	(1,216)
Age	43.93	39.86	-2.06**	43.04	37.70	-2.38**
	(16.83)	(18.62)	(1,372)	(17.41)	(17.82)	(1,232)
Education	3.74	3.61	-1.49	3.73	3.82	1.04
	(0.46)	(0.56)	(1,331)	(0.46)	(0.39)	(1,198)
Income	5.76	4.62	-2.35**	3.99	3.29	-1.60
	(2.58)	(2.50)	(1,259)	(2.12)	(1.88)	(1,117)
Not in work	0.14	0.11	-0.70	0.19	0.11	-1.55
	(0.35)	(0.32)	(1,379)	(0.39)	(0.31)	(1,234)
Trust in elected politicians						
Gender	0.50	0.57	0.80	0.50	0.71	2.60***
	(0.50)	(0.50)	(1,344)	(0.50)	(0.46)	(1,216)
Age	43.84	40.94	-1.37	42.87	40.42	-1.00
	(16.84)	(18.98)	(1,372)	(17.41)	(18.72)	(1,232)
Education	3.73	3.75	0.15	3.73	3.82	0.83
	(0.46)	(0.44)	(1,331)	(0.46)	(0.39)	(1,198)
Income	5.73	5.61	-0.20	3.98	3.69	-0.49
	(2.57)	(2.38)	(1,259)	(2.11)	(2.81)	(1,117)
Not in work	0.15	0.03	-2.71***	0.19	0.09	-1.69*
	(0.35)	(0.17)	(1,379)	(0.39)	(0.30)	(1,234)
Government competency						
Gender	0.50	0.62	1.51	0.50	0.61	1.31
	(0.50)	(0.49)	(1,344)	(0.50)	(0.49)	(1,216)
Age	43.64	44.77	0.56	42.81	41.69	-0.45
	(16.83)	(19.06)	(1,372)	(17.43)	(18.41)	(1,232)
Education	3.74	3.63	-1.20	3.73	3.87	1.14
	(0.46)	(0.49)	(1,331)	(0.46)	(0.35)	(1,198)
Income	5.74	5.12	-1.20	3.98	3.58	-0.65
	(2.59)	(2.35)	(1,259)	(2.11)	(2.23)	(1,117)
Not in work	0.15	0.04	-2.58**	0.19	0.10	-1.59
	(0.35)	(0.20)	(1,379)	(0.39)	(0.30)	(1,234)

Notes: Table reports the mean values for respondents and non-respondents of our main outcome variables. 'Gender' takes the value of 1 for female; 'age' runs from 18 to 89 (continuous); 'education' ranges from 1 to 4; 'income' ranges from 1 to 10; 'not in work' takes the value of 1 when people are not in paid work or on furlough. Asterisks indicate significant differences in mean values between samples from a Wald test of significance (with degrees of freedom in parentheses). Standard deviations are below the means, in parentheses. *** p<0.01, ** p<0.05 , * p<0.1.

S14 Table reports balance tests between respondents and non-respondents of our main variables of interest. The total number of non-respondents which we have demographic data for is very small: 35 in the US and 38 in the UK

taking 'trust in government' and gender as an example. The table illustrates that there are hardly any significant differences between respondents and non-respondents. Non-respondents of our trust in government variable are somewhat younger in both countries and of slightly lower income in the US. There are no consistent differences across countries for either of the other two outcome variables. Overall, the low non-respondent numbers and lacking consistent differences between respondents and non-respondents are reassuring for the validity of our main results.
