# Supplementary material for: Good news reduces trust in government and its efficacy: The case of the Pfizer/BioNTech vaccine announcement
Source: PLoS One. 2021 Dec 9;16(12):e0260216. doi: 10.1371/journal.pone.0260216 (PMC8659308; doi:10.1371/journal.pone.0260216)
Supplement: S15 Table — (ZIP) [file pone.0260216.s015.zip › s15_table.pdf]

S15 Table. Treatment effects of vaccine announcement using Edelman data

	Trust in various institutions (US)				
	Government	Media	Business	NGOs	Employer
Vaccine	-1.010**	-0.500	-0.576	-0.322	0.571
	(0.478)	(0.455)	(0.419)	(0.466)	(0.594)
Time	0.080***	0.064***	0.038*	0.027	-0.014
	(0.024)	(0.023)	(0.021)	(0.024)	(0.033)
Observations	1,077	1,077	1,077	1,077	503

Notes: Each estimate comes from an individual linear regression. The trust variables all range from 1 (Do not trust them at all) to 9 (Trust them a great deal). All estimates are weighted based on the general population weight included in the Edelman dataset. *** $p<0.01$, ** $p<0.05$, * $p<0.1$.

S15 Table reports the results of the main analysis using the October/November 2020 Edelman Trust Barometer. The Edelman dataset includes a total of 2,612 US respondents with with 1,345 questioned prior to the vaccine announcement, and 910 questioned after the announcement. The overall fieldwork period lasted from the October 19 to November 18, 2020. We test the effect of the vaccine announcement on all trust variables available in the Edelman dataset and find that only trust in government was negatively and significantly affected by the vaccine announcement. The magnitude of the effect is also sizeable: Post-vaccine announcement, trust in government is reduced by 1 point on a 10-point scale. The results also indicate that the overall time trend in governmental trust was weakly positive at the time of the vaccine announcement.
