# Supplementary material for: Good news reduces trust in government and its efficacy: The case of the Pfizer/BioNTech vaccine announcement
Source: PLoS One. 2021 Dec 9;16(12):e0260216. doi: 10.1371/journal.pone.0260216 (PMC8659308; doi:10.1371/journal.pone.0260216)
Supplement: S1 Fig — (ZIP) [file pone.0260216.s016.zip › s1_fig.pdf]

S1 Fig. Media attention in the US and the UK

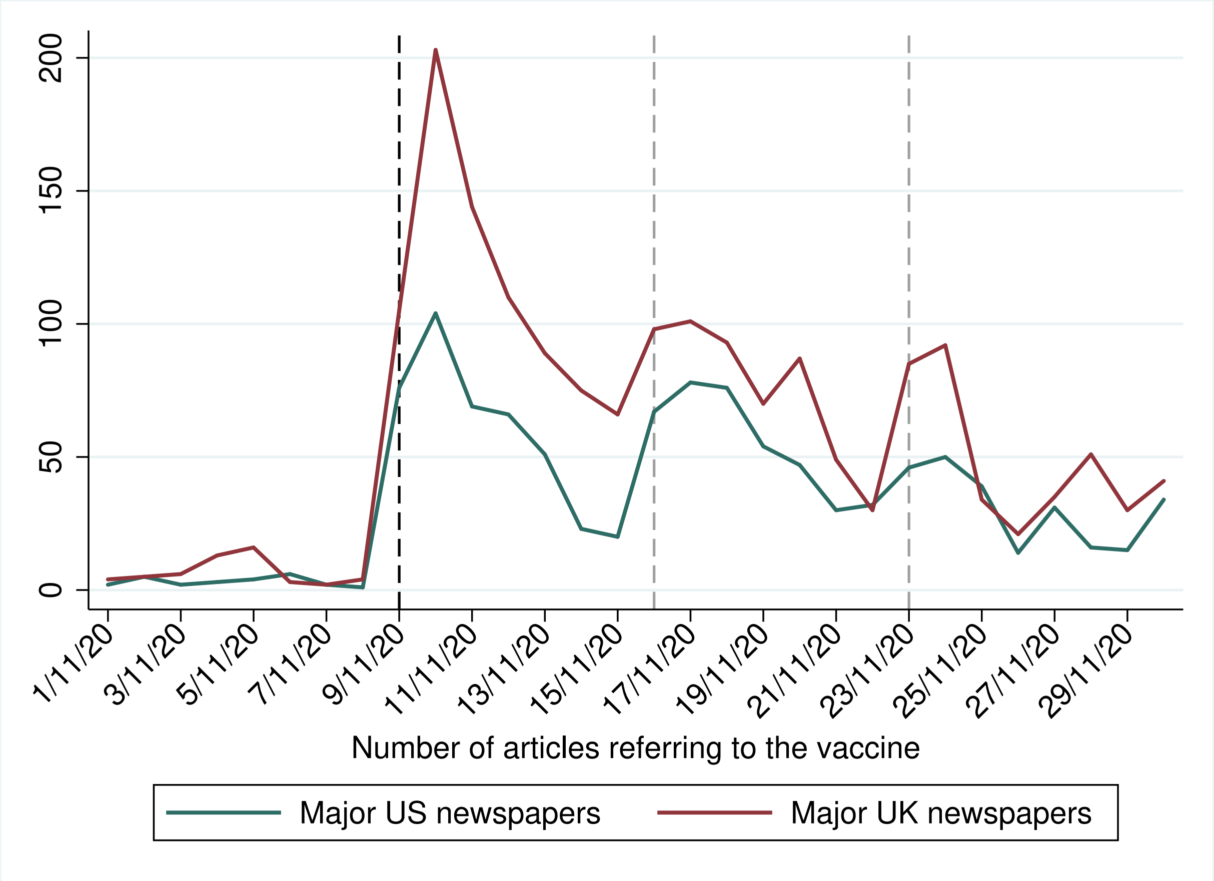

Source: Factiva Global News Database
Notes: The figure shows the daily number of articles referring to 'vaccine' and 'Pfizer', in newspapers that are part of the Factiva categories 'Top US Newspapers' and 'Top UK Newspapers'. Duplicates and republished news are excluded. The reference lines are for the announcement of the vaccine developed by Pfizer/BioNTech (in black), and the later announcements by Moderna and Oxford University/AstraZeneca, respectively (both in gray).

S1 Fig. shows that there was a huge amount of attention for the vaccine in the main newspapers in the US and UK. The number of articles referring to 'vaccine' and 'Pfizer' peaked on November 10, 2020 – the day after the Pfizer/BioNTech announcement – and stayed relatively high until late November, not only because of the initial announcement, but also because of subsequent announcements of vaccine success by Moderna (November 16) and Oxford University/AstraZeneca (November 23).
