# Supplementary material for: Good news reduces trust in government and its efficacy: The case of the Pfizer/BioNTech vaccine announcement
Source: PLoS One. 2021 Dec 9;16(12):e0260216. doi: 10.1371/journal.pone.0260216 (PMC8659308; doi:10.1371/journal.pone.0260216)
Supplement: S2 Fig — (ZIP) [file pone.0260216.s017.zip › s2_fig.pdf]

S2 Fig. US search interest in the presidential elections

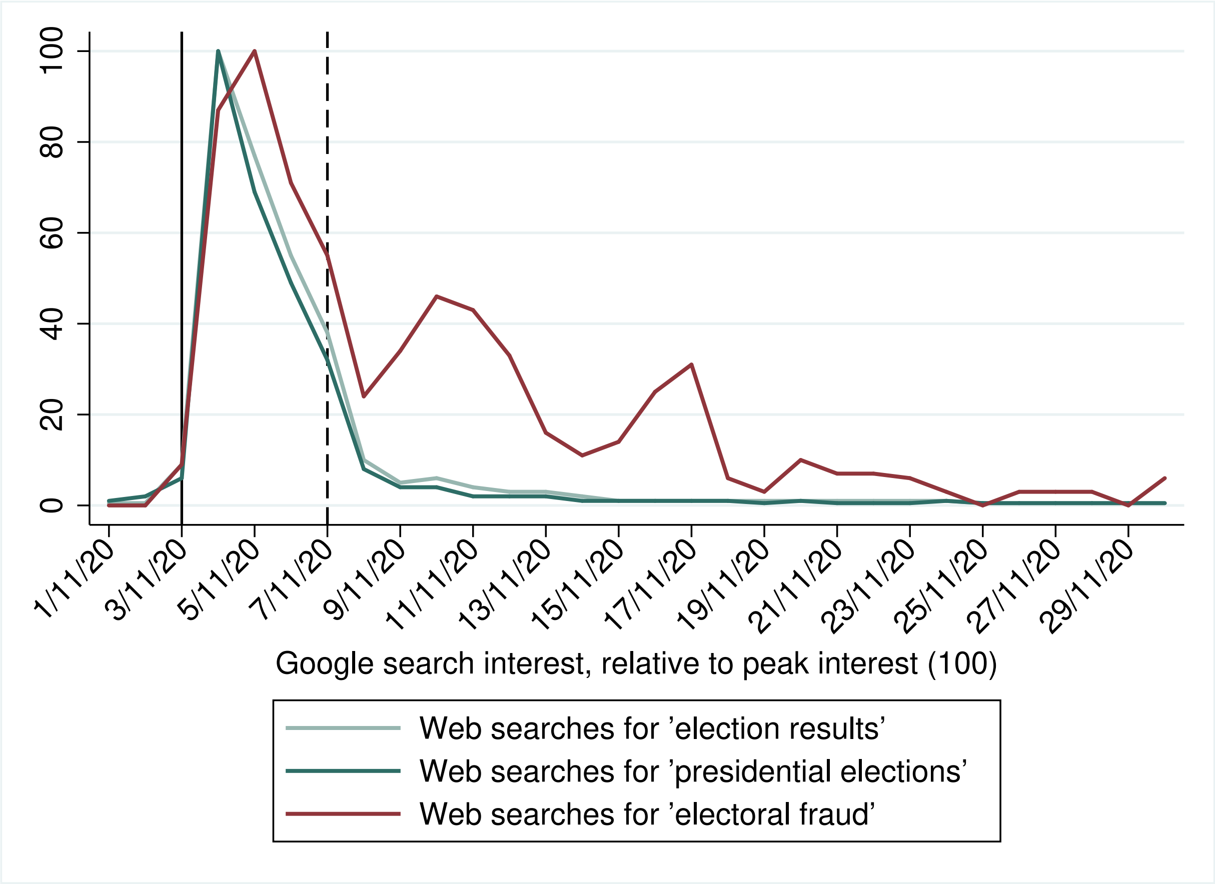

Source: Google Trends

Notes: The numbers represent search interest relative to the highest point in the figure, for the United States in the period from November 1, 2020 to November 30, 2020. For instance, a value of 50 means that the relevant search term is half as popular.
