# Supplementary material for: Good news reduces trust in government and its efficacy: The case of the Pfizer/BioNTech vaccine announcement
Source: PLoS One. 2021 Dec 9;16(12):e0260216. doi: 10.1371/journal.pone.0260216 (PMC8659308; doi:10.1371/journal.pone.0260216)
Supplement: S1 Appendix — (ZIP) [file pone.0260216.s018.zip › s1_appendix.pdf]

S1 Appendix. Coincidental and collateral events

Coincidental events

The Pfizer/BioNTech vaccine announcement did not coincide with other key (political) events. The week before our pre-treatment survey wave had seen several critical events. In the US, the 2020 Presidential Elections had taken place on November 3, with the results taking a (relatively) long time to materialize. Joe Biden's win was announced by all major news networks on 7 November, even though some uncertainty about state-level results remained throughout our survey period. This is captured in S2 Fig, which shows that US search interest in the elections had already decreased by the time that the results were called by the networks, and had further declined to very low levels by the time that the vaccine was announced. By contrast, levels of interest in 'electoral fraud' remained higher, even though they also declined over time. The period did not see any concession by the sitting president, Donald Trump.

In the UK, a second nation-wide lockdown had been announced on November 1, and had been in place since November 5. Though the introduction of lockdowns has been associated with an increase in trust in government [1], it is, in principle, possible that frustration with the new lockdown built up over the days, and (only) found expression in post-announcement government evaluations. However, our evidence suggests this was not the case. S4 Table shows the impact of the vaccine announcement on citizens' assessment of three aspects of lockdown measures: their introduction, easing, and severity. The highest values on these variables reflect the most positive evaluations, while low values indicate that respondents either considered the measures too slow (too relaxed) or too quick (too severe). In the (locked-down) UK, post-announcement lockdown evaluations were not significantly more negative than pre-announcement evaluations, except for the evaluations of the introduction of lockdowns by the at-risk subgroup. By contrast, in the US, where no changes took place in (nation-wide) lockdown measures, evaluations among the post-announcement group were, in some cases, significantly more negative.

Collateral events

Though the Pfizer/BioNTech vaccine announcement was the key event in our survey period, it triggered 'collateral events' that are relevant for government evaluations. In particular, while politicians were careful not to claim direct credit for the vaccine, they did respond in ways that were meant to reflect well on their own performance.

In the US, then Vice-President Mike Pence immediately stated that the development of the vaccine had benefited from the administration's Operation Warp Speed Program; a claim that was later rejected by Pfizer/BioNTech [2]. President Donald Trump's initial response was based on the same assumption that governments benefit from (good) vaccine news; yet, it took the form of fury with the timing of the announcement (just after the Presidential Elections), which meant that his chances of being re-elected had not been boosted [3]. He changed his tone later that week by

expressing high levels of optimism about the development of vaccine candidates and about the speed of the vaccines' roll-out [4].

Meanwhile, in the UK, politicians responded to the news by (cautiously) expressing high hopes for the future. Prime Minister Boris Johnson referred to the 'scientific cavalry', and said he was 'buoyantly optimistic about the prospects of this country next year' [5]. Moreover, both the Prime Minister and Health Secretary Matt Hancock immediately started emphasising that they had already purchased 40 million Pfizer/BioNTech vaccine doses, that the UK's vaccine approval process would be efficient, and that UK citizens would be among the first to receive the vaccine [6-8]. Finally, commentators clearly expected the news to have a positive effect on the government and prime minister. For instance, one headline read: 'The Covid-19 vaccine could be just what Boris needs to save his premiership' [9].

We should emphasize that if these 'collateral events' had impacted on government evaluations, the effect would have been the reverse; that is, we should have seen a *positive* rather than negative effect on the evaluations. Instead, despite these events, confidence in government, and politicians more generally, declined.

References

1. Bol, D, Giani, M, Blais, A, Loewen, PJ. The effect of COVID-19 lockdowns on political support: Some good news for democracy? Eur. J. Polit. Res. 2021; 60: 497-505.
2. R. Griffin, R, Armstrong, D. Pfizer vaccine's funding came from Berlin, not Washington. The Boston Globe. 2020 Nov 9 [Cited 2021 May 28].
3. Johnson, CY, Abutaleb, Y, McGinley, L, Dawsey, J. Pfizer's post-election day vaccine report rankles Trump. The Washington Post 2020 Nov 12 [Cited 2021 May 28].
4. Haberman, M, Grady, D. Trump projects a rosy view on vaccines, while Biden blasts the federal coronavirus response. The New York Times Online. 2020 Nov 13 [Cited 2021 May 28].
5. Donnelly, L. A great day for humanity. The Daily Telegraph. 2020 Nov 10 [Cited 2021 May 28].
6. Swinford, S. Covid can be beaten but don't drop our guard, warns Jonathan Van-Tam. The Times. 2020 Nov 9 [Cited 2021 May 28].
7. Stephens, M. British public to be among first to receive Covid-19 vaccine. The Telegraph Online. 2020 Nov 10 [Cited 2021 May 28].
8. Anderson, C. Boris says Britain is FRONT of queue for coronavirus vaccine in 'SIGNIFICANT' breakthrough. The Express Online. 2020 Nov 9 [Cited 2021 May 28].
9. Clark, R. The Covid-19 vaccine could be just what Boris needs to save his premiership. The Telegraph Online. 2020 Nov 9 [Cited 2021 May 28].
