# Supplementary material for: Good news reduces trust in government and its efficacy: The case of the Pfizer/BioNTech vaccine announcement
Source: PLoS One. 2021 Dec 9;16(12):e0260216. doi: 10.1371/journal.pone.0260216 (PMC8659308; doi:10.1371/journal.pone.0260216)
Supplement: S2 Appendix — (ZIP) [file pone.0260216.s019.zip › s2_appendix.pdf]

S2 Appendix. Description of main variables

Trust in government (D18 US/ D17 UK). Variable capturing how much of the time respondent i believes she can trust the federal government in Washington/Westminster.

1: Hardy ever

2: Some of the time

3: Most of the time

4: Just about always

Trust in elected politicians (D19 US/ D18 UK). Variable capturing how much trust respondent i has in elected politicians in general.

1: None at all

2: A little

3: Some

4: Quite a bit

5: A lot

Government competency (D30 US/ D29 UK). Variable capturing how competent respondent i assesses the government's response to COVID-19.

1: Very incompetent

2: Incompetent

3: Somewhat competent

4: Competent

5: Very competent

Seriousness. (P1) Variable capturing respondent i's assessment of the seriousness of COVID-19 compared to the seasonal flu.

1: Not at all serious

2: Not very serious

3: Fairly serious

4: Very serious

Concern/anxiety.(P2) Variable capturing respondent i's concern for themselves and their family due to COVID-19.

1: Not at all concerned

2: Not very concerned

3: Fairly concerned

4: Very concerned

Economic concern.(P3) Variable capturing respondent i's concern about the economic implications of COVID-19.

1: Not at all concerned

2: Not very concerned

3: Fairly concerned

4: Very concerned

Compliance.(P4) Variable capturing how likely respondent i is to follow the government's guidance for reducing the spread of COVID-19.

1: Very unlikely

2: Fairly unlikely

3: Neither likely nor unlikely

4: Fairly likely

5: Very likely

Others follow guidelines.(D32 US/ D31 UK) Variable capturing how likely respondent i believes it to be that other people will follow government guidance related to reducing the spread of COVID-19.

1: Very unlikely

2: Fairly unlikely

3: Neither likely nor unlikely

4: Fairly likely

5: Very likely

Luck vs. effort.(D10 US/ D9 UK) Variable ranging from 0 to 10, capturing whether respondent i believes income differences arise primarily from luck (0) or effort (10).

Willingness to pay.(Part 1) Variable ranging from 0 £/$ to above £200/$260 capturing how much respondent i would be willing to pay for a treatment to reduce own mortality risk from COVID-19, based on Part I of the survey instrument.

Religiosity.(D22 US/ D21 UK) Categorical variable capturing how religious respondent i considers herself to be.

1: Not religious at all

2: Not very religious

3: Fairly religious

4: Very religious

Party (UK). Categorical variable capturing which party respondent i feels closest to.

1: Conservative

2: Labour

3: Liberal Democrat

4: Scottish National Party (SNP)

5: Plaid Cymru

6: The Brexit Party

7: Green Party

8: United Kingdom Independence Party (UKIP)

9: Democratic Unionist Party

10: Sinn Fein

11: Social Democratic and Labour Party (SDLP)

12: Alliance Party

13: Ulster Unionist Party

14: Other

Party (US). Categorical variable capturing which party respondent i feels closest to.

1: Democratic Party

2: Republican Party

3: Other

Education (UK). Categorical variable capturing respondent i's highest level of educational attainment.

1: No formal education

2: Primary education

3: Secondary education

4: Higher education and above

Education (US). Categorical variable capturing respondent i's highest level of educational attainment.

1: No formal education

2: Elementary school

3: High school

4: College and above

Gender. Binary variable coded as 1 if subject i indicated to be female, 0 if subject i indicated to be male. Subjects who indicated "other" or "prefer not to say" were coded as missing values (n=22).

Age. Categorical variable capturing the age bracket of subject i.

1: 18-20 years old

2: 21-29 years old

3: 30-39 years old

4: 40-49 years old

5: 50-59 years old

6: 60 years or older

Left-right. Categorical variable capturing how much subject i agrees with the statement: "On economic policy matters, there is a role for the government".

1: Strongly disagree

2: Disagree

3: Neither agree nor disagree

4: Agree

5: Strongly agree

Sample. Categorical variable indicating whether subject i is a resident in the US or UK.

1: United States

2: United Kingdom

Income. Categorical variable capturing the income bracket of subject i. Values are stated in Pound Sterling (£) for subjects from the UK and in US Dollars ($) for subjects from the US.

1: Under 10,000

2: 10,000 to 20,000

3: 20,001 to 30,000

4: 30,001 to 40,000

5: 40,001 to 50,000

6: 50,001 to 60,000

7: 60,001 to 80,000

8: 80,001 to 100,000

9: 100,001 to 150,000

6: Above 150,000
