# Supplementary material for: Good news reduces trust in government and its efficacy: The case of the Pfizer/BioNTech vaccine announcement
Source: PLoS One. 2021 Dec 9;16(12):e0260216. doi: 10.1371/journal.pone.0260216 (PMC8659308; doi:10.1371/journal.pone.0260216)
Supplement: S3 Appendix — (ZIP) [file pone.0260216.s020.zip › s3_appendix.pdf]

S3 Appendix. Survey instrument

United States

Part I – Willingness to pay elicitation

Consider two hypothetical treatments for COVID-19 that must be taken when healthy: Treatment 1 and Treatment 2. When you take either treatment your chances of dying from COVID-19 over the next 3 months fall by the same amount: the survival rates improve by the equivalent of 5 people in 1 million. That is, if in a population of a million people everyone took the treatment, then, on average we expect that 5 people within that population will survive who would have died from COVID-19 over the next 3 months. But we cannot identify which 5 people.
Treatment 1 contributes to reducing your chances of death because it aids recovery if you get serious COVID-19 and are admitted to hospital. It does not affect your chances of getting COVID-19 or transmitting it to others.
Treatment 2 contributes to reducing your chances of death because it reduces the likelihood that you will become infected by COVID-19 and hence also that you will transmit the infection to others.

How much would you pay to receive treatment 1?

- $0
- $32.5
- $65
- $97.5
- $130
- $162.5
- $195
- $227.5
- $260
- Above $260

How much would you pay to receive treatment 2?

- $0
- $32.5
- $65
- $97.5
- $130
- $162.5
- $195
- $227.5

- $260
- Above $260

Part II – Perceptions

Please answer the following questions about the spread of the coronavirus COVID-19.

P1: How serious do you think COVID-19 is compared to the seasonal flu?

- Not at all serious
- Not very serious
- Fairly serious
- Very serious
- Don't know

P2: How concerned are you for you and your family about COVID-19?

- Not at all concerned
- Not very concerned
- Fairly concerned
- Very concerned
- Don't know

P3: How concerned are you about the economic implications of COVID-19?

- Not at all concerned
- Not very concerned
- Fairly concerned
- Very concerned
- Don't know

P4: How likely are you to follow government's guidance for reducing the spread of COVID-19?

- Very unlikely
- Fairly unlikely
- Neither likely nor unlikely
- Fairly likely
- Very likely
- Don't know

K1: How many people in the US would you estimate will have died in total due to coronavirus by the end of 2020?

K2: By what percentage would you estimate average income in the US will be lower in 2020 as compared to 2019?

Part III – Demographic questions

D1: Which US state do you live in?

D2: Are you Spanish, Hispanic, or Latino?

- Yes
- No

D3: Below you will find a list of five race categories. Please choose one or more races that you consider yourself to be:

- White
- Black or African-American
- American Indian or Alaska Native
- Asian
- Native Hawaiian or other Pacific Islander
- Other group
- Prefer not to answer

D4: What is your household income before tax?

- Under $10,000
- $10,000 - $20,000
- $20,001 - $30,000
- $30,001 - $40,000
- $40,001 - $50,000
- $50,001 - $60,000
- $60,001 - $80,000
- $80,001 - $100,000
- $100,001 - $150,000
- $150,001 - $200,000
- Above $200,000
- Don't know
- Prefer not to answer

D5: Which party do you feel closest to?

- Democratic Party
- Republican Party
- Other
- Don't know

D6: Thinking about the 2016 Presidental Election, to your best recollection, whom did you vote for?

- Hillary Clinton
- Donald Trump
- Other candidate
- Didn't vote
- Don't know
- Prefer not to say

D6.1: Thinking about the recent 2020 Presidental Election, whom did you vote for?

- Joe Biden
- Donald Trump
- Other candidate
- Didn't vote
- Don't know
- Prefer not to say

D7: In politics people sometimes talk of left and right. Where would you place yourself on the following scale?

[Scale from 0 (Left) to 10 (Right)]

D8: Some people feel that government should make much greater efforts to make people's incomes more equal. Other people feel that government should be much less concerned about how equal people's incomes are. Where would you place yourself on this scale?

[Scale from 0 (Try to make incomes equal) to 10 (Be less concerned about equal incomes)]

D9: Some people think that society would be a better place if people had more respect for authority. Other people think society would be a better place if people questioned authority more often. Where would you place yourself on this scale?

[Scale from 0 (Respect authority) to 10 (Question authority)]

D10: To what extent do you believe that income differences arise from luck and to what extent from differences in effort and skills? [Scale from 0 (From luck) to 10 (From effort and skills)]

D11: To what extent do you think it is acceptable for income differences to exist if they arise from luck? [Scale from 0 (Not acceptable at all) to 10 (Completely acceptable)]

D12: To what extent, if at all, would you support the government introducing a Universal Basic Income, where the government makes sure that everyone has an income, without a means test or requirement to work?

- Very supportive
- Supportive
- Neither supportive or unsupportive
- Unsupportive

- Very unsupportive
- Don't know

D13: How, if at all, has your support for Universal Basic Income changed due to the economic impact of the COVID-19 pandemic?

- Much more supportive
- Somewhat more supportive
- Neither more or less supportive
- Somewhat less supportive
- Much less supportive
- Don't know

D14: Do you think that the federal government in Washington could be doing more to tackle climate change, or is it already doing as much as it reasonably can?

- Could be doing more
- Doing as much as it reasonably can
- Don't know

D15: To what extent, if at all, would you support the government introducing more extensive policies to tackle climate change?

- Very supportive
- Supportive
- Neither supportive or unsupportive
- Unsupportive
- Very unsupportive
- Don't know

D16: During the last seven days, on average how much time (if any) have you spent per day following the news?

- None, no time at all
- Less than 1/2 hour
- 1/2 hour to 1 hour
- 1 to 2 hours
- More than 2 hours
- Don't know

D17: Generally speaking, would you say that most people can be trusted, or that you can't be too careful in dealing with people?

- Most people can be trusted
- Can't be too careful
- Don't know

D18: How much of the time do you think you can trust the federal government in Washington to do what is right?

- Hardly ever
- Some of the time
- Most of the time
- Just about always
- Don't know

D19: How much trust do you have in elected politicians in general?

- None at all
- A little
- Some
- Quite a bit
- A lot
- Don't know

D20: Which of these best describes what you were doing last week?

- Working full time (30 or more hours per week)
- Working part time (8-29 hours a week)
- Working part time (less than 8 hours a week)
- On furlough (temporary leave)
- Unemployed and looking for work
- Full time university student
- Other full time student
- Retired
- Not in paid work for any other reason
- Other

D21: What is your highest level of educational attainment?

- College and above
- High school
- Elementary school
- No formal education

D22: How religious do you consider yourself to be?

- Very religious
- Fairly religious
- Not very religious
- Not religious at all

- Don't know
- Prefer not to say

D22.1: During the next months, how likely or unlikely is it that you will not have enough money to cover your day to day living costs?

- Very unlikely
- Fairly unlikely
- Neither likely nor unlikely
- Fairly likely
- Very likely
- Don't know

D23: Thinking about the past month, did you, as a result of the COVID-19 pandemic, earn less, about the same or more money than usual?

- Less than usual
- About the same
- More than usual
- Don't know

D24: How healthy have you felt in the last weeks?

[Scale from 0 (Not healthy at all) to 10 (Very healthy)]

D25: According to US government guidelines, those above the age of 65 and/or those with underlying health conditions are at an increased risk from COVID-19. Do you consider yourself to be in this group?

- Yes
- No
- Don't know
- Prefer not to say

D26: How likely or unlikely do you think it is that you have had the coronavirus?

- Very unlikely
- Fairly unlikely
- Neither likely nor unlikely
- Fairly likely
- Very likely
- Don't know

D27: How would you assess the government's introduction of lockdown measures?

- Too quick
- Fairly quick

- About right
- Fairly slow
- Too slow
- Don't know

D28: How would you assess the government's relaxation of lockdown measures?

- Too quick
- Fairly quick
- About right
- Fairly slow
- Too slow
- Don't know

D29: How would you assess the severity of the government's lockdown measures?

- Too severe
- Severe
- About right
- Relaxed
- Too relaxed
- Don't know

D30: How competent would you assess the government's response to COVID-19?

- Very competent
- Competent
- Somewhat competent
- Incompetent
- Very incompetent
- Don't know

D31: To what extent do you think you can influence the likelihood of catching COVID-19 through your own behavior and actions?

- Not at all
- Somewhat
- Quite a bit
- A lot
- Don't know

D32: Do you think other people are likely to comply with the government's guidance for reducing the spread of COVID-19?

- Very unlikely
- Fairly unlikely
- Neither likely nor unlikely
- Fairly likely
- Very likely
- Don't know

D33: Please tell us, in general, how willing or unwilling you are to take risks. Please use a scale from 0 to 10, where 0 means "completely unwilling to take risks" and a 10 means you are "very willing to take risks". You can also use any number between 0 and 10 to indicate where you fall on the scale.

[Scale from 0 (Completely unwilling to take risks) to 10 (Very willing to take risks)]

D34: Are you generally an impatient person, or someone who always shows great patience? Please use a scale from 0 to 10 where 0 means "very impatient" and a 10 means you are "very patient". You can also use any numbers between 0 and 10 to indicate where you fall on the scale.

[Scale from 0 (Very impatient) to 10 (Very patient)]

D35: Imagine you were given $10 to divide between yourself and another person in increments of $1. Considering your current situation, how much of the $10 would you keep for yourself and how much would you give to the other person? Please use the slider below to indicate how much you would give to the other person.

D36: Imagine you won $1,000 in a lottery. Considering your current situation, how much would you donate to charity?

Feedback: Please let us know in the field below whether you have any feedback regarding the study. Were any of the questions or tasks unclear?

United Kingdom

Part I – Willingness to pay elicitation

Consider two hypothetical treatments for COVID-19 that must be taken when healthy: Treatment 1 and Treatment 2. When you take either treatment your chances of dying from COVID-19 over the next 3 months fall by the same amount: the survival rates improve by the equivalent of 5 people in 1 million. That is, if in a population of a million people everyone took the treatment, then, on average we expect that 5 people within that population will survive who would have died from COVID-19 over the next 3 months. But we cannot identify which 5 people.

Treatment 1 contributes to reducing your chances of death because it aids recovery if you get serious COVID-19 and are admitted to hospital. It does not affect your chances of getting COVID-19 or transmitting it to others.

Treatment 2 contributes to reducing your chances of death because it reduces the likelihood that you will become infected by COVID-19 and hence also that you will transmit the infection to others.

How much would you pay to receive treatment 1?

- £0
- £25
- £50
- £75
- £100
- £125
- £150
- £175
- £200
- Above £200

How much would you pay to receive treatment 2?

- £0
- £25
- £50
- £75
- £100
- £125
- £150
- £175
- £200
- Above £200

Part II – Perceptions

Please answer the following questions about the spread of the coronavirus COVID-19.

P1: How serious do you think COVID-19 is compared to the seasonal flu?

- Not at all serious
- Not very serious
- Fairly serious
- Very serious
- Don't know

P2: How concerned are you for you and your family about COVID-19?

- Not at all concerned
- Not very concerned
- Fairly concerned
- Very concerned
- Don't know

P3: How concerned are you about the economic implications of COVID-19?

- Not at all concerned
- Not very concerned
- Fairly concerned
- Very concerned
- Don't know

P4: How likely are you to follow government's guidance for reducing the spread of COVID-19?

- Very unlikely
- Fairly unlikely
- Neither likley nor unlikely
- Fairly likely
- Very likely
- Don't know

K1: How many people in the UK would you estimate will have died in total due to coronavirus by the end of 2020?

K2: By what percentage would you estimate average income in the UK will be lower in 2020 as compared to 2019?

Part III - Demographic questions

D1: Which area of the United Kingdom do you live in?

- England
- Scotland
- Wales
- Northern Ireland

D2: To which of these groups do you consider you belong?

- White British
- Any other white background
- White and Black Caribbean
- White and Black African

- White and Asian
- Any other mixed background
- Indian
- Pakistani
- Bangladeshi
- Chinese
- Any other Asian background
- Black Carribean
- Black African
- Any other black background
- Other ethnic group
- Prefer not to answer

D3: What is your household income before tax?

- Under £10,000
- £10,000 - £20,000
- £20,001 - £30,000
- £30,001 - £40,000
- £40,001 - £50,000
- £50,001 - £60,000
- £60,001 - £80,000
- £80,001 - £100,000
- £100,001 - £150,000
- Above £150,000
- Don't know
- Prefer not to answer

D4: Which party do you feel closest to?

- Conservative
- Labour
- Liberal Democrat
- Scottish National Party (SNP)
- Plaid Cymru
- The Brexit Party
- Green Party
- United Kingdom Independence Party (UKIP)

- Democratic Unionist Party
- Sinn Fein
- Social Democratic and Labour Party (SDLP)
- Alliance Party
- Ulster Unionist Party
- Other
- Don't know

D5: Thinking about the 2016 Brexit referendum, to your best recollection, which side did you vote for, 'Leave', or 'Remain'?

- Leave
- Remain
- Didn't vote
- Don't know
- Prefer not to say

D6: In politics people sometimes talk of left and right. Where would you place yourself on the following scale?

[Scale from 0 (Left) to 10 (Right)]

D7: Some people feel that government should make much greater efforts to make people's incomes more equal. Other people feel that government should be much less concerned about how equal people's incomes are. Where would you place yourself on this scale?

[Scale from 0 (Try to make incomes equal) to 10 (Be less concerned about equal incomes)]

D8: Some people think that society would be a better place if people had more respect for authority. Other people think society would be a better place if people questioned authority more often. Where would you place yourself on this scale?

[Scale from 0 (Respect authority) to 10 (Question authority)]

D9: To what extent do you believe that income differences arise from luck and to what extent from differences in effort and skills?

[Scale from 0 (From luck) to 10 (From effort and skills)]

D10: To what extent do you think it is acceptable for income differences to exist if they arise from luck?

[Scale from 0 (Not acceptable at all) to 10 (Completely acceptable)]

D11: To what extent, if at all, would you support the government introducing a Universal Basic Income, where the government makes sure that everyone has an income, without a means test or requirement to work?

- Very supportive
- Supportive
- Neither supportive or unsupportive

- Unsupportive
- Very unsupportive
- Don't know

D12: How, if at all, has your support for Universal Basic Income changed due to the economic impact of the COVID-19 pandemic?

- Much more supportive
- Somewhat more supportive
- Neither more or less supportive
- Somewhat less supportive
- Much less supportive
- Don't know

D13: Do you think that the government in Westminster could be doing more to tackle climate change, or is it already doing as much as it reasonably can?

- Could be doing more
- Doing as much as it reasonably can
- Don't know

D14: To what extent, if at all, would you support the government introducing more extensive policies to tackle climate change?

- Very supportive
- Supportive
- Neither supportive or unsupportive
- Unsupportive
- Very unsupportive
- Don't know

D15: During the last seven days, on average how much time (if any) have you spent per day following the news?

- None, no time at all
- Less than 1/2 hour
- 1/2 hour to 1 hour
- 1 to 2 hours
- More than 2 hours
- Don't know

D16: Generally speaking, would you say that most people can be trusted, or that you can't be too careful in dealing with people?

- Most people can be trusted

- Can't be too careful
- Don't know

D17: How much of the time do you think you can trust the Westminster government to do what is right?

- Hardly ever
- Some of the time
- Most of the time
- Just about always
- Don't know

D18: How much trust do you have in elected politicians in general?

- None at all
- A little
- Some
- Quite a bit
- A lot
- Don't know

D19: Which of these best describes what you were doing last week?

- Working full time (30 or more hours per week)
- Working part time (8-29 hours a week)
- Working part time (less than 8 hours a week)
- On furlough (temporary leave)
- Unemployed and looking for work
- Full time university student
- Other full time student
- Retired
- Not in paid work for any other reason
- Other

D20: What is your highest level of educational attainment?

- Higher Education and above
- Secondary education
- Primary education
- No formal education

D21: How religious do you consider yourself to be?

- Very religious
- Fairly religious

- Not very religious
- Not religious at all
- Don't know
- Prefer not to say

D22.old: During the next months, how likely or unlikely is it that you will not have enough money to cover your day to day living costs?

- Very unlikely
- Fairly unlikely
- Neither likely nor unlikely
- Fairly likely
- Very likely
- Don't know

D22: Thinking about the past month, did you, as a result of the COVID-19 pandemic, earn less, about the same or more money than usual?

- Less than usual
- About the same
- More than usual
- Don't know

D23: How healthy have you felt in the last weeks?

[Scale from 0 (Not healthy at all) to 10 (Very healthy)]

D24: According to UK government guidelines, those above the age of 70 and/or those with underlying health conditions are at an increased risk from COVID-19. Do you consider yourself to be in this group?

- Yes
- No
- Don't know
- Prefer not to say

D25: How likely or unlikely do you think it is that you have had the coronavirus?

- Very unlikely
- Fairly unlikely
- Neither likely nor unlikely
- Fairly likely
- Very likely
- Don't know

D26: How would you assess the government's introduction of lockdown measures?

- Too quick
- Fairly quick
- About right
- Fairly slow
- Too slow
- Don't know

D27: How would you assess the government's relaxation of lockdown measures?

- Too quick
- Fairly quick
- About right
- Fairly slow
- Too slow
- Don't know

D28: How would you assess the severity of the government's lockdown measures?

- Too severe
- Severe
- About right
- Relaxed
- Too relaxed
- Don't know

D29: How competent would you assess the government's response to COVID-19?

- Very competent
- Competent
- Somewhat competent
- Incompetent
- Very incompetent
- Don't know

D30: To what extent do you think you can influence the likelihood of catching COVID-19 through your own behavior and actions?

- Not at all
- Somewhat
- Quite a bit
- A lot
- Don't know

D31: Do you think other people are likely to comply with the government's guidance for reducing the spread of COVID-19?

- Very unlikely
- Fairly unlikely
- Neither likely nor unlikely
- Fairly likely
- Very likely
- Don't know

D32: Please tell us, in general, how willing or unwilling you are to take risks. Please use a scale from 0 to 10, where 0 means "completely unwilling to take risks" and a 10 means you are "very willing to take risks". You can also use any number between 0 and 10 to indicate where you fall on the scale.

[Scale from 0 (Completely unwilling to take risks) to 10 (Very willing to take risks)]

D33: Are you generally an impatient person, or someone who always shows great patience? Please use a scale from 0 to 10 where 0 means "very impatient" and a 10 means you are "very patient". You can also use any numbers between 0 and 10 to indicate where you fall on the scale.

[Scale from 0 (Very impatient) to 10 (Very patient)]

D34: Imagine you were given £10 to divide between yourself and another person in increments of £1. Considering your current situation, how much of the £10 would you keep for yourself and how much would you give to the other person? Please use the slider below to indicate how much you would give to the other person.

D35: Imagine you won £1,000 in a lottery. Considering your current situation, how much would you donate to charity?

Feedback: Please let us know in the field below whether you have any feedback regarding the study. Were any of the questions or tasks unclear?
